# Recent Advances in Graphene-Based Nanocomposites for Ammonia Detection

**DOI:** 10.3390/polym14235125

**Published:** 2022-11-24

**Authors:** Sara Maira M. Hizam, Adel Mohammed Al-Dhahebi, Mohamed Shuaib Mohamed Saheed

**Affiliations:** 1 Centre of Innovative Nanostructures and Nanodevices (COINN), Universiti Teknologi PETRONAS, Seri Iskandar 32610, Perak, Malaysia; 2 Department of Fundamental and Applied Sciences, Universiti Teknologi PETRONAS, Seri Iskandar 32610, Perak, Malaysia; 3 Department of Mechanical Engineering, Universiti Teknologi PETRONAS, Seri Iskandar 32610, Perak, Malaysia

**Keywords:** graphene oxide, reduced graphene oxide, polymer nanocomposites, polymeric matrices, hazardous gas, sensing mechanism

## Abstract

The increasing demand to mitigate the alarming effects of the emission of ammonia (NH_3_) on human health and the environment has highlighted the growing attention to the design of reliable and effective sensing technologies using novel materials and unique nanocomposites with tunable functionalities. Among the state-of-the-art ammonia detection materials, graphene-based polymeric nanocomposites have gained significant attention. Despite the ever-increasing number of publications on graphene-based polymeric nanocomposites for ammonia detection, various understandings and information regarding the process, mechanisms, and new material components have not been fully explored. Therefore, this review summarises the recent progress of graphene-based polymeric nanocomposites for ammonia detection. A comprehensive discussion is provided on the various gas sensor designs, including chemiresistive, Quartz Crystal Microbalance (QCM), and Field-Effect Transistor (FET), as well as gas sensors utilising the graphene-based polymer nanocomposites, in addition to highlighting the pros and cons of graphene to enhance the performance of gas sensors. Moreover, the various techniques used to fabricate graphene-based nanocomposites and the numerous polymer electrolytes (e.g., conductive polymeric electrolytes), the ion transport models, and the fabrication and detection mechanisms of ammonia are critically addressed. Finally, a brief outlook on the significant progress, future opportunities, and challenges of graphene-based polymer nanocomposites for the application of ammonia detection are presented.

## 1. Introduction

Graphene originates from the word “graphite”, while the suffix “-ene” reflects the allotrope of carbon that consists of stacked graphene layers. This form of carbon derivative consists of two-dimensional (2D) sheets of sp^2^ hybridised carbon atoms arranged in a hexagonal lattice extended to a honeycomb-shaped network with other essential allotropes. Graphene has been considered an excellent nanofillers for constructing high performance composite materials due to its unique 2D structure and the remarkable physicochemical properties. Among its exceptional properties include the high mechanical strength with a high Young’s modulus of 1 TPa and a high tensile stress of 130 GPa [1], an excellent thermal conductivity of up to approximately ~50,000 W/mK [2], a high mobility of approximately 1400 cm^2^/Vs, which is higher compared to those of the broadly used semiconductors and silica [3,4], a large surface area that could reach up to 2630 m^2^/g [5], and thermal stability [2,4]. Graphene is also extremely light, with its density estimated to be as low as 1.06 g/cm^2^. In view of this, graphene has shown tremendous potential to improve the properties of sensing and biosensing devices. Thus, graphene-based materials, such as Graphene Oxide (GO) and reduced Graphene Oxide (rGO), have been extensively utilised in ammonia gas sensing due to the fact of their unique structures and functionalities [6,7,8]. Graphene-based polymeric nanocomposites possess enhanced physical and mechanical properties, which makes them appealing for various applications, such as sensors, batteries, e-textiles, and wearable electronics. The addition of graphene in a polymer matrix has been utilised to achieve such a remarkable improvement in terms of sensitivity, wide detection range and selectivity criteria of sensors and biosensing devices [9,10,11]. The challenge of obtaining this remarkable achievement is that many parameters, such as the type of graphene used, the orientation of the graphene layers, and the choice of preparation method, play an crucial role in stipulating the properties of graphene-polymeric composites [12,13].

**Figure 1:**
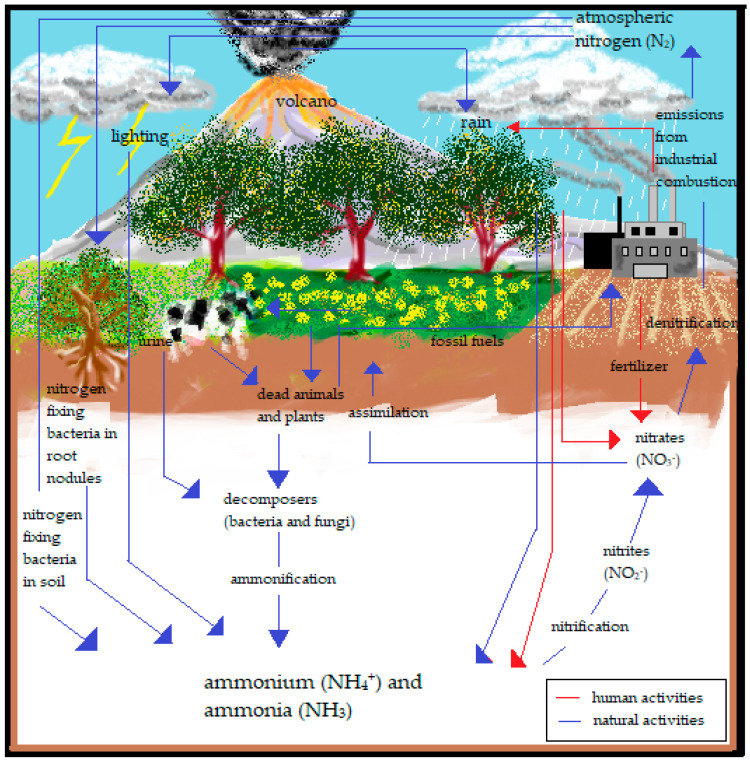
Nitrogen cycle of ammonia through the biosphere.

Ammonia (NH_3_) gas is one of the largest produced toxic Volatile Organic Compounds (VOCs) in the industrial sector, apart from benzene, toluene, and others. It is a ubiquitous gas that is produced naturally in soils from bacterial metabolism and the decaying processes of plants, animals, and animal wastes. It can also be observed in the natural environment, such as in soil, air, and water, due to the involvement of NH_3_ in the nitrogen cycle, as shown in Figure 1. NH_3_ has been broadly utilised in the production of explosives, fertilisers, plastics, fabrics, pesticides, dyes, and as an industrial coolant. Nevertheless, the widespread use of NH_3_ on farms, such as in fertilizer to cultivate soil and farmland, in cleaning products for households, and in industrial and commercial applications, such as glass cleaning, cooking grease solution, wine stain beakers, waste and wastewater treatment, cold storage, and stabilisers, signifies a frequent exposure of NH_3_ through accidental discharge, erosion, mechanical failure, construction defect, nitrification by nitrogen-fixing bacteria, and combustion of fossil fuels in both chemical and transportation industries [14,15,16]. Hence, their overwhelming production should be monitored to control the pollutants and avoid any catastrophic occurrence, such as explosions and long exposure to the environment, which would certainly lead to detrimental results. 

Generally, NH_3_ gas does not settle in low-lying areas since it is lighter than air and often forms a vapour in the presence of moisture that rapidly scatters in the form of fog. The distinctive effects of NH_3_ exposure on humans greatly depend on the concentration of NH_3_, as shown in Table 1. Inhalation of only a small dose of NH_3_ vapour may cause severe health effects and fatal poisoning in humans [15,17]. An ammonia molecule has a lone pair located at the nitrogen atom, which makes the molecule a strong electron acceptor and is categorised in the electron-withdrawing group. However, NH_3_ reacts with oxygen ions on the surface of a metal oxide and donates electrons by returning to the trapped electrons, as expressed in Equations (1)–(3) [18,19]. Usually, the reactions occur on the surface in a humid atmosphere. Nevertheless, the reactions that take place in the presence of 60–72% of relative humidity do not influence the sensing performance of the sensor [18].

2NH_3_ + 3O^−^ _(adsorbed)_ → N_2_ + 3H_2_O + 3e^−^ (1)

2NH_3_ + 4O^−^ _(adsorbed)_ → N_2_O + 3H_2_O + 4e^−^ (2)

2NH_3_ + 5O^−^ _(adsorbed)_ → 2NO + 3H_2_O +5e^−^ (3)

Sensors are analytical tools, which consist of an active sensing material with a signal transducer, that detect changes in their environment and send the information to other electronics, usually a computer processor, for data acquisition and interpretation. The first developed sensors for the detection of organic vapours, methanol, and formaldehyde were introduced by Sumner et al. (1923) [21]. To date, the fabrication of gas sensors using graphene-based polymeric nanocomposites has emerged as a promising novel class of materials owing to the high specific area, controlled interfacial interactions, greater achievable loads, and higher overall compliances. The synergistic effects (the interfacial interactions) between graphene-based materials and polymer matrices play an essential role in improving the sensitivity of gas sensing devices. The development of graphene-based sensors is ascribed to several distinctive features, such as a large surface-to-volume ratio, eccentric optical properties, remarkable carrier mobility, and exceptional electrical and thermal properties. Despite the multiple reports and publications regarding the advancement and recent applications of carbon-based NH_3_ gas sensors, research on graphene-based sensors is still ongoing and various aspects, including the process, the mechanism of sensors, and new materials, have not been explored extensively. Recently, Tang et al. (2021) reported the development and progression of functionalised graphene sensors for NH_3_ detection at room temperature (RT) and its sensing mechanism using graphene and other nanoparticles and polymers. Several challenges that hinder the mass production of such sensors were highlighted along with a number of proposals that address these problems as well as the potential opportunities and prospective applications of graphene-based NH_3_ sensors [7]. In another report, Bannov et al. (2021) discussed the recent advances in carbon-based NH_3_ gas sensors, including GO, graphene, carbon nanofibers and related materials. The paper discussed the sensing characteristics of carbon nanomaterials-based gas sensors, analysed the various techniques of NH_3_ gas sensors, the problems related to the sensors recovery, and the effect of relative humidity on the sensing behaviour of carbon nanomaterials [22]. Another study by Gopinath et al. (2020) reviewed the various applications of carbon-based materials, which emphasised the adsorption of toxic gases and the removal of pollutants from ecosystems via numerous carbon nanomaterials, such as biochar, activated carbon, Carbon Nanotubes (CNTs), and graphene. The authors also stressed the application of carbon materials and the advantages of the addition of biochar [23].

Based on the discussion above, the fabrication of graphene-based polymeric nanocomposite remains a challenge mainly attributed to the agglomeration of graphene 2D materials particularly at higher concentrations. To overcome this, several solution processing techniques can be used to achieve the homogenous distribution of graphene within the polymeric matrices. In addition, the synthesis of high-quality graphene plays an important role in the dispersion and, hence, improves the final sensitivity of the sensor. In an attempt to provide a comprehensive insight of the recent progress of graphene-based polymeric nanocomposites, this review was conducted. It summarises the latest updates on the use of graphene-based polymer nanocomposites in gas sensing applications, specifically its latest application, gas sensor development, synthesis of graphene, and mechanism. While various past reviews have addressed graphene-based polymer nanocomposites, this paper particularly discusses the recent advances in graphene-based polymer nanocomposites for NH_3_ gas sensor application, which includes a thorough explanation of the principle and designs of gas sensors, a summary of the role of graphene-based materials for enhancing a gas sensor’s performance for NH_3_ detection based on recent studies, and the synthesis of graphene-based materials. The diverse range of methods used to fabricate NH_3_-based graphene–polymer nanocomposites is also critically addressed. In addition, this review provides an extensive analysis of the mechanism of graphene-based polymer nanocomposites and the various polymer electrolytes applied for NH_3_ detection. Finally, this review concludes by addressing the future perspectives and challenges regarding gas sensors for a safe and sustainable environment.

## 2. Structural Properties and Synthesis of Graphene

**Figure 2:**
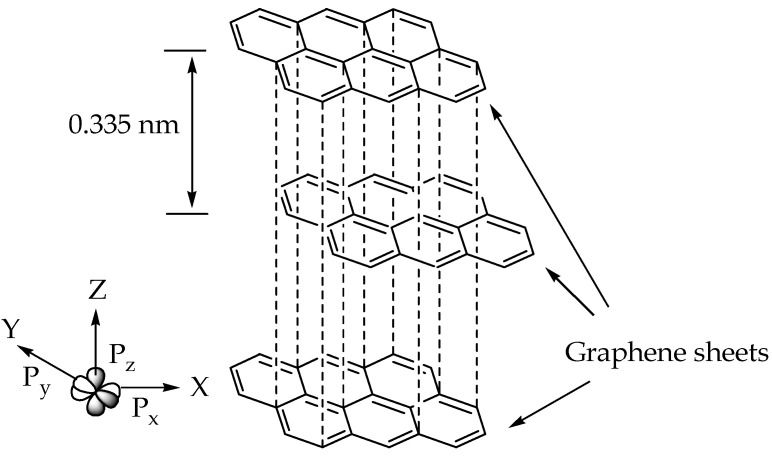
The layered sp^2^ hybridised carbon atoms in a hexagonal lattice graphene structure.

Graphene is an allotrope of carbon that consists of unique hybridisation properties [24,25]. Generally, carbon has a ground state orbital configuration of 1s^2^ 2s^2^ 2p^2^ in which the energy gaps between the 2s and 2p orbitals are narrow, promoting one 2s electron to jump to a vacant higher energy 2p orbital. The electron excitation allows carbon to hybridise into sp, sp^2^, and sp^3^ configurations, leading to a variety of molecular structures. Every orbital configuration has a specific molecular geometry. For instance, the sp orbital configuration forms circular regions or nodes to represent the s orbital and the dumbbell structure of the p_x_, p_y_, and p_z_ orbital, which forms chain structures. Meanwhile, the sp^2^ and sp^3^ orbitals form planar and tetrahedral structures, respectively. All graphene derivatives exhibit the sp^2^ and sp structure due to the fact of their hexagonal ring structure with layers of the crystalline honeycomb lattice. The 2p_z_ orbital of the carbon atoms can imbricate successfully if they are parallel or out-of-plane π bonds, contributing to the lowest energy of the graphene sheet when it is completely flat. Furthermore, the π orbital in graphene located specifically at the double-bonded C=C region is scattered throughout the graphene sheet, making it highly mobile and thermally and electrically conductive. The distribution of π orbital is known as the delocalisation of electrons. The hexagonal lattice with π electrons tends to undergo electron delocalisation to stabilise the force due to the spreading energy over a larger area rather than confining it to a small area. The graphene layers in graphite are usually separated by a minimal gap of 0.335 nm from each other [26], as shown in Figure 2. A weak van der Waals forces holds the adjacent graphene sheets together, allowing them to move easily and become more lubricative towards one another.

**Figure 3:**
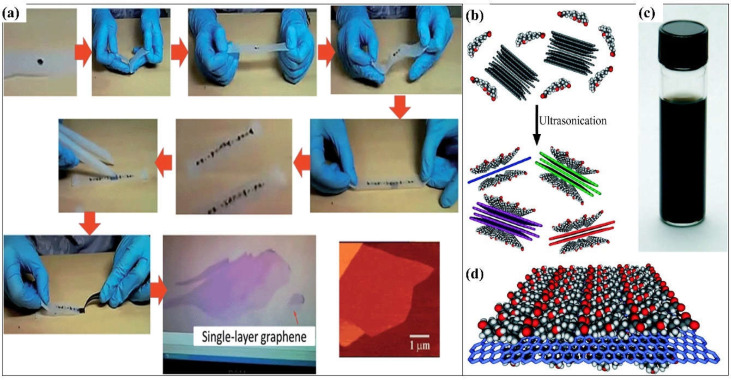
Schematic illustration of graphene synthesis methods: (**a**) Scotch tape method to produce single-layer graphene via the cleavage of HOPG sample. Adapted with permission from [28]. Copyright RSC 2015. (**b**) Schematic illustration of the exfoliation process of graphene via the ultrasonication of graphite flakes with sodium cholate (SC). (**c**) Optical image of six-week dispersion in SC. (**d**) Schematic illustration of the ordered SC monolayer on graphene. Adapted with permission from [29]. Copyright ACS 2009.

Since its discovery in 2004, graphene and its derivatives have been constantly studied for their synthesis, functionalities, and applications. Over the years, various methods have been developed to synthesize graphene layers. For example, the mechanical exfoliation, or Scotch tape method, is a very straightforward process to produce graphene layers. Adhesive tape is the most important material needed for this method (Figure 3a). Basically, the graphite crystals of the graphite flakes are attached to the tape. Several peel offs are necessary to obtain multiple layers of graphene, as can be observed under a microscope. Depending on the preparation of the wafer, each peeling exhibits a different size and thickness of the graphene layer. Figure 3 illustrates the graphene layer synthesis and the exfoliation process using the Scotch tape method. The epitaxial growth method consists of two approaches: silicon carbide (SiC) crystal and nickel (Ni) diffusion methods. The epitaxial growth method using SiC crystals is a simple method in which a graphene monolayer or bilayer is grown on the surface of the SiC crystals after a heating and cooling process [27]. Several parameters affect the growth of the graphene layer, such as the temperature, pressure, and heating rate. The graphene converts into a nanotube under uncontrollable temperature and pressure The graphene layers grow simultaneously at random places on the surface of the SiC crystals, while the Ni diffusion method almost resembles the aforementioned method. The Ni surface also has a similar lattice structure to graphene, with a lattice constant of approximately 98.7%. Hence, a thin layer of the Ni layer is first evaporated onto the SiC crystals. Next, the formation of graphene or graphite layer on the surface of the SiC crystals are caused by the diffusion of through the Ni layer. The attached graphene layer on the surface of the SiC crystals via this method is stronger than that of the previous method. 

**Figure 4:**
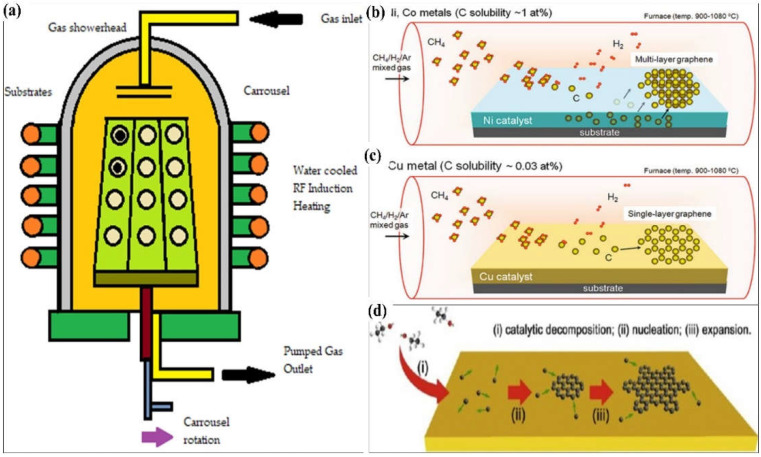
Schematic illustration of (**a**) epitaxial growth systems. CVD growth of graphene sheets on different metals: (**b**) Ni and Co. (**c**) Single-layer graphene growth. Adapted with permission from [32]. Copyright Springer 2015. (**d**) Large-scale CVD growth of graphene on Cu foil. (**i**) catalytic decomposition, (**ii**) nucleation, and (**iii**) expansion. Adapted with permission from [33]. Copyright Elsevier 2015.

Chemical Vapour Deposition (CVD) is another essential method that can produce high-quality graphene layers. Figure 4 shows the epitaxial growth system and the CVD growth system used to produce graphene on Ni, cobalt (Co), and copper (Cu). CVD is a more notable and optimal method that involves the exposure of a substrate to the gas containing various functional groups, for example, amine, methane (CH_4_), hydrogen, and argon. A square inch of metal, such as Ni, Cu, Co, platinum (Pt), or iridium (Ir), is placed in a quartz-tube furnace. The metal is coated with sustainable and waste materials, such as butter, camphor (C_10_H_16_O), tea tree extraction (*Melaleuca Alternifoliate*), waste plastic in solid form, cookies, and chocolates [30,31]. Without a catalyst or filament, the plasma is used to grow a thin film and allow the by-products to evaporate. At an elevated temperature of approximately 800–1100 °C, the gas deposits the carbon onto the surface of the metal. A graphene monolayer or multilayer grows on the surface after a prolonged cooling down in an inert gas atmosphere. Graphene is stamped onto the required substrate after the metal has been etched, the polymer support peels off afterwards.

Generally, GO consists of carbon allotropes with hydroxyl and epoxy groups on the interior side and edges of the structure, making it more hydrophilic. This would also provide GO with high solubility and reactive sites for the nucleation and growth of polymer in many organic solvents, especially water, leading to the formation of graphene-based hybrids. Nevertheless, the defection of the graphene is directly proportional to the electrical conductivity, which would support the widening application of GO in electrical materials and devices. The reduction process would increase the amount of unsaturated carbon, resulting in conjugation among benzene structures and a high electrical conductivity [34,35]. GO can be produced from graphite via the infamous Hummers Offeman, Brodie, and Staudenmaeir methods. A bulk of graphite sheet can be delaminated into individual graphene sheets under typical mechanical actions due to the overlapping of p_z_ orbitals and intense interactions of neighboring graphene layers. In addition, GO can be synthesised via the self-assembly process and hydrothermal method. Meanwhile, GO can be reduced to form rGO using several methods including the mechanical exfoliation of a single sheet of graphene film, CVD of graphene monolayers, epitaxial growth of graphene films, chemical or thermal reduction of graphene derivatives, and longitudinal “unzipping” of CNTs [36,37].

Liquid phase exfoliation is also known as graphite oxide exfoliation which usually originates from graphite oxide before obtaining GO and finally rGO (Figure 3b). The well-known hydrophilic nature of graphite oxide has made it easier to disperse in water via sonication or stirring. After being well-dispersed, strong acid and oxidiser are added and mixed well in the dispersed graphite oxide solution to obtain GO. Next, a reducing agent is added to obtain the rGO. Various methods have been reported to obtain rGO, including mechanical exfoliation of natural graphite, CVD, epitaxial growth, longitudinal “unzipping” of CNTs, reduction of graphene derivatives, and liquid-phase exfoliation. Each of the aforementioned methods has distinctive techniques and results, which are applicable for specific applications, including sensors, electronics, and solar cells. Thus, selecting the right method for the rGO fabrication should be given the highest priority. The chemical and thermal reduction processes are the most favourable methods to synthesis graphene extensively. The elimination of oxygenated-functional groups of GO, such as hydroxyl, epoxy, carbonyl, and carboxyl groups, can be executed using reducing agents, such as hydrazine, ascorbic acid [38,39,40], oxalic acid, glucose [41,42], and pyrrole [43,44,45,46,47,48]. This method produces a more defected rGO in the presence of dangling oxygenated-functional groups on the interior and graphene structural molecules on the edge. The most defected rGO plays a crucial role in the detection of several organic compounds. This method is more controllable as the elimination of oxygen-containing functional groups is highly dependent on the experimental parameters, such as the temperature, reduction time, type of reducing agent, and environment of reduction. Table 2 shows the distinctive advantages and limitations of graphene-based materials synthesis methods.

The remarkable properties of graphene nanocomposites, including the large specific surface area and excellent conductivity, contribute to improving the ions’ mobility and, thus, the sensitivity of the sensors. Graphene nanomaterials provide excellent surface functionalities to various materials including fibres, films, etc. which improve the electroactivity of these composites. According to recent studies, graphene can detect not only well-known toxic gases such as NH_3_, nitrogen dioxide (NO_2_), carbon monoxide (CO), sulphur dioxide (SO_2_), and others, but also chemical warfare agents [64,65,66,67,68,69]. Chemical warfare agents, such as tabun (GA), sarin (GB), soman (GD), cyclosarin (GF), novichok, and R-VX and VX, are extremely toxic synthetic chemicals which disperses as a gas, liquid, or aerosol or as an agent that absorbs to particles to become a powder. A significant contribution to the bond formation between toxic gas and graphene is highly attributed to the functional groups of graphene-possessing carboxyl, hydroxyl, and amine located at the edge of graphene surface. Upon detection, due to the bond formation, the structural changes can be observed through X-ray Photoelectron Spectroscopy (XPS), Fourier Transform Infrared (FTIR), Nuclear Magnetic spectroscopy (NMR), Ultraviolet-Visible (UV-Vis), and Thermogravimetric Analysis (TGA) [67,68]. Despite the fact that the chemical structure of graphene can be varied, it can be tuned by controlling their size, shape, graphene surface, and charge transfer between functional groups and by doping with heteroatoms [70]. Additionally, understanding the chemical interactions between graphene-based polymeric composites play an important role in improving the synthetic strategies for designing novel graphene nanocomposites with tunable functionalities and superior sensing performance. The following sections discuss the various methods used for the design of graphene-based polymeric nanocomposites for the detection of gases and chemical warfare.

## 3. Fabrication Methods of Graphene-Based Polymer Nanocomposites

**Figure 5:**
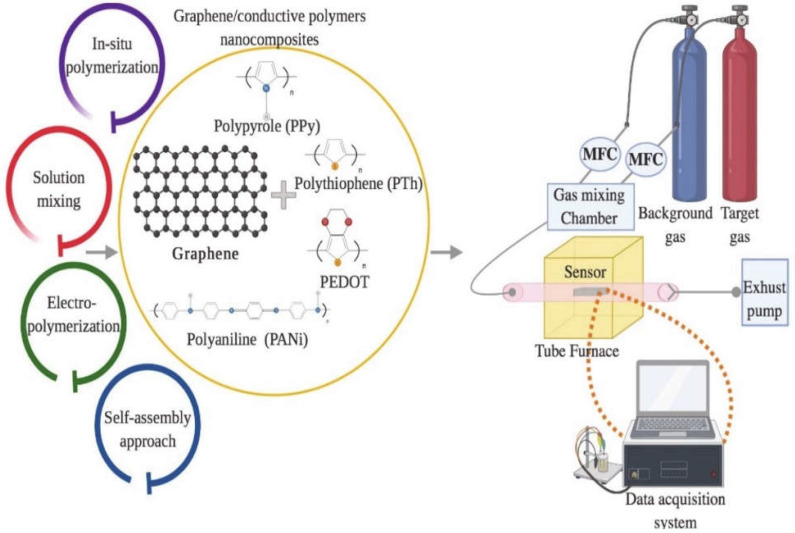
Schematic diagram for the preparation of graphene/conductive polymer composites and their applications for gas sensing. Adapted with permission from [82]. Copyright MDPI 2020.

Graphene/polymer nanocomposites can be fabricated through several methods, namely, in situ polymerisation, melt intercalation, electropolymerisation, self-assembly, and solution mixing [71,72], as portrayed in Figure 5. In the in situ polymerisation technique, the graphene fillers are mixed in the presence of catalysts, followed by polymerisation with a head start of heating or radiation [73]. This technique forms a firm interaction between the filler and the polymer matrix, rapid stress transfer, expeditious formation of homogeneous distribution, and remarkable miscibility for a higher percentage of filler material in the composites. However, the drawbacks of this method include the elevated viscosity of the solution as well as the formation of aggregates, which make processing harder, restricting the addition of filler and requiring the removal of the solvent to obtain the composites when the use of solvent is compulsory during the process. Meanwhile, the graphene is combined with a polymer matrix in the molten state without the use of solvent at elevated temperatures in the melt intercalation method [74]. The advantage of the melt intercalation technique is that the process involves a solvent-free reaction and is compatible for use in the preparation of the thermoplastic composite. The only drawback of this method is the development of poor diversity and distribution in the matrix, apart from the need for high shear forces to break and defect the graphene sheets.

Solution mixing on the other hand is an effective method to form composite graphene-based polymer composites. In this method, re the polymer is well-mixed in a solvent with graphene nanomaterials leading to an increase in the curvature of its curvilinear surface, the dispersity, and the solubility of the graphene [75]. However, it is necessary to functionalise the graphene sheets to facilitate the dispensability in different solvents. Thus, the graphene must be completely dissolved in a solvent to avoid aggregation. Although this method can be applied for large-scale production and does not need special equipment, the graphene can easily aggregate once the solvent is evaporated. Therefore, the risk of aggregation can be minimised by utilising the power and melting blending for low filler material or solution blending for high filler material. The electrospinning technique is one of the potential method to fabricate graphene-based polymer nanocomposites, which includes the electrification of a liquid droplet to generate a jet [76]. Then, the liquid becomes more viscous, and it is stretched out from a metallic needle directly to a ground conductive metal collecting screen, which is wrapped with aluminium alloy foil [77,78,79,80]. This technique is also very simple, cost effective, versatile, and widely used to impregnate graphene within the polymer matrices and along the fibre axis [81]. However, good conductive graphene–polymer composites can only be created when the graphene solution is uniformly dispersed into the polymer matrices. Conversely, an inhomogeneous solution may form weak molecular interactions, which may reduce the graphene loading capacity and alter the properties of the solution. Figure 5 shows the synthesis of graphene-based conductive polymers and their gas sensing applications.

**Figure 6:**
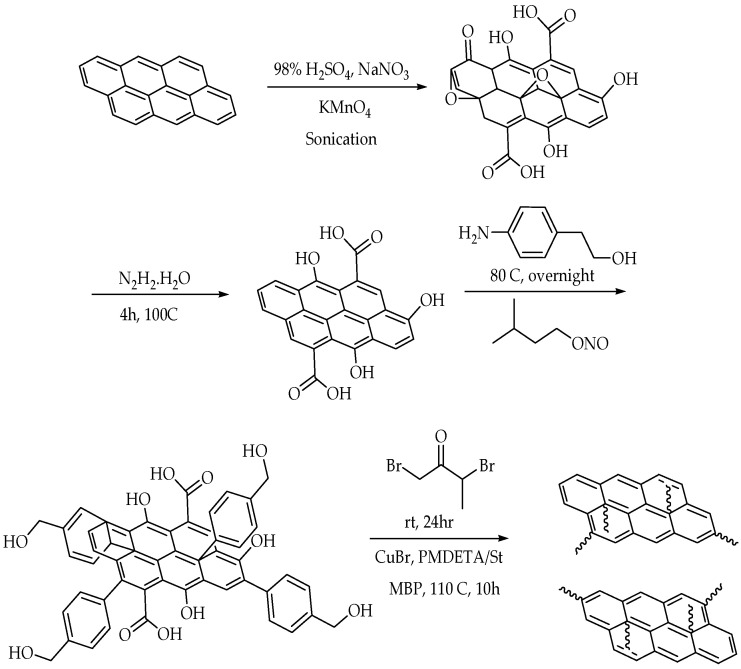
Schematic illustration of the fabrication of polystyrene-functionalised graphene nanosheets. H_2_SO_4_: sulphuric acid; NaNO_3_: sodium nitrate; KMnO_4_: potassium permanganate; N_2_H_2_.H_2_O: hydrazine monohydrate; NH_2_(C_6_H_4_)CH_2_CH_2_OH: 2-(4-aminophenyl)ethanol; CH_3_CH_2_(CH_3_)CH_2_CH_2_ONO: isoamyl nitrite; BrCH_2_COCH(Br)CH_3_: 2-bromopropionyl bromide; CuBr: copper bromide; PMDETA: N,N,N’,N’,N”-pentamethyl-diethylenetriamine; MBP: 2-bromopropionate [88].

The most commonly used graphene-based reinforcement materials include polycarbonate, polyester, epoxy, polyethene, polypropylene, polystyrene, polymethylmethacrylate, nylon, and Teflon [71,83]. The polarity, molecular weight, graphene functionalities, hydrophobicity, and solvent interaction highly influence the mechanism of the graphene-based polymer nanocomposites [84,85,86]. During the mixing of graphene and polymer, the ratio of graphene to polymer and the molecular weight of the grafting polymer usually play an important role in the dispersion of graphene [87]. Since the graphene itself has multiple layers, the predicted size ranges between 20 and 40 nm. Meanwhile, the polymer, for instance, polystyrene, has a molecular weight of approximately 60,000 g/mol. Reducing the graphene nanosheet size would decrease the interlayer cohesive energy and, thus, reduce the viscosity of the solution, which limits the introduction of excess free volume in the solvent system. Figure 6 shows an example of the fabrication of polystyrene-functionalised graphene nanosheets. Atom Transfer Radical Polymerisation (ATRP) is used to control the thickness constraint and molecular structure of the graphene host interface.

Polymers’ functionalised graphene nanofillers have received significant traction in the nanofabrication of polymer composites due their unique properties, such as mechanical, optical, magnetic, and electronic properties [89]. The interactions that occur between the graphene-based materials and the polymer matrices are grouped into covalent and noncovalent interactions or bonds. These interactions usually involve weak bonds, for instance, dipole–dipole moments, van der Waals forces (or London dispersion forces), and hydrogen forces, between two electronegative atoms with almost similar electronegativity. Upon these interactions, graphene-based nanocomposites consist of three different structures, namely, phase-separated, intercalated, and exfoliated. The phase-separated graphene composite is a simple method that comprises two steps, which are the reduction of graphene and the chemical deposition of graphene into the reduced graphene. Phase-separated structures can be observed via the colour, texture, and phase difference [90,91,92].

On the contrary, intercalated graphene occurs when fabricated materials/atoms are inserted between the graphene sheet layers. The intercalation structure prevents the agglomeration of the material. The addition of graphene sheets also assists in providing active sites and retaining the size growth of the material. Pure graphene sheets with great π-π interaction tend to collapse with each other. However, the intercalation structure can reduce the π-π stacking interaction between graphene sheets, making it favourable for three-dimensional (3D) mesoporous structures [93,94]. Meanwhile, exfoliated graphene is usually synthesised through the mechanical exfoliation of graphite using the Scotch tape method, as shown in Figure 3. Basically, the graphene appears as a single layer with reduced defects (I_D_/I_G_ = 0.25 or less) and a high carbon-to-oxygen (C/O) ratio (>20) compared to rGO and electrochemical exfoliated graphene [95]. Regardless of the high exfoliation efficiency (>90%), the exfoliation statistically yields very low single-layer graphene (up to 5%). Despite their low yield, this method could produce high-quality and selective graphene layers without the need to add intercalants, chemicals, or solvents. Exfoliated graphene can be strengthened using a DC plasma spray setup integrated with a custom-designed inert atmosphere shroud [95].

The exceptional properties of graphene highly contribute to the development of optical electronics [96], composites [97], photovoltaic systems [98], sensors [99], and dye solar cells [100]. Although graphene has many variations, such as CNTs, MXene, fullerene, and other hexagonal lattices carbon-based compounds, the properties of graphene are matchless, and the potential of graphene is still widely explored. While CNTs have a higher Young’s modulus and tensile strength compared to other existing fillers [26], the extraordinary properties of graphene, such as its high carrier mobility and quantum Hall effect at RT, have surpassed the capability of CNTs. Therefore, graphene demonstrates an undoubtful potential for the fabrication of gas sensors due to the fact of its exceptional device features, such as miniature, low-cost portable characteristics, and formidable sensing performance. The involvement of graphene in developing gas sensors is ascribed to several distinctive unique features, such as a large surface-to-volume ratio, high carrier mobility, and outstanding electrical and thermal properties as compared to other carbon allotropes. The large surface area of graphene leads to an incredible transport capability and an extremely small band gap that assists the loadings of the desired molecules, leading to an interaction between the analyte molecule and electrode surface. Graphene is also well known for its high LOD, excellent sensing range, short signal response, and good reproducibility, which make graphene an exceptional sensor platform. Interestingly, the low environmental impact caused by graphene has made it highly environmentally friendly in the development of gas sensors.

## 4. Graphene Nanocomposite-Based NH_3_ Sensors

Graphene nanocomposites based NH_3_ gas sensors possess several unique structural functionalities that enables the absorbance of gaseous molecules from the environment with improved sensing capabilities, such as low Limit of Detection (LOD), rapid response time, reproducibility, and stability. Among the proposed methods to effectively fabricate NH_3_ gas sensors using graphene-based polymer nanocomposites include sol-gel, hydrothermal or solvothermal, layer-by-layer deposition, template-assisted deposition, and physical vapour deposition. The aforementioned methods have been used to fabricate graphene-based compounds that contribute to its wide range of applications. Table 3 summarizes the advantages and disadvantages of each method.

### 4.1. Sol-Gel Method

The sol-gel method is a wet chemistry-based synthesis route that involves the solidification of a compound containing a highly chemically active component through a solution in the form of sol or gel under mild conditions, followed by heat treatment. The method has recently been applied to fabricate glass, oxide coatings, and functional ceramic powders, especially high critical-temperature oxide superconductors and composite oxide materials that are complicated to prepare through conventional methods. Generally, the technique employs the hydrolysis and polycondensation of alkoxide-based metal precursors, for instance R_4-n_SiX_n_ compounds (n = 1–4, X = OR’). In the organic route, “sol” is obtained by dissolving an alkoxide precursor in a specific solvent, meanwhile “gel” is acquired by the supervised addition of gelatine agents, such as water, that is initially under acidic or basic conditions to initiate the condensation reactions that lead to the formation of a 3D oligomeric network. Apart from the aforementioned addition process, the formation of a gel can be formed by the condensation process, solvent evaporation, and syneresis of a species. The transformation of films on the sensor substrate from the resultant gel is possible via dipping, spin-coating, or spraying techniques. The deposited film is attached to the substrate by treating it with a low-temperature annealing process and t prolonged heating to evaporate all organic residues and water molecules. The reaction starts with solvation, where the metal cation, M^z+^, attracts water molecules to form the solvent of M (H_2_O)_n_^z+^ and is highly inclined to release proton (H^+^) to maintain the equilibrium.

M (H_2_O)_n_^z+^ → M(H_2_O)_n−1_(OH)_z−1_ + H^+^ (4)

The next step in the sol-gel method is the hydrolysis reaction. The nonionising molecular precursors, such as metal alkoxide (M(OR)_n_), react with water.

M(OR)_n_ + xH_2_O → M(OR)_n−x_(OH)_x_ (5)

M(OR)_n−x_(OH)_x_ + xROH → M(OH)_n_ (6)

In the final step, the polycondensation reaction takes place, which depends on the type of removed molecules that gives off two routes of reactions: dehydration polycondensation and dealcoholisation polycondensation.

Dehydration polycondensation: -M-OH + HO-M- → -M-O-M- H2O(7)

Dealcoholisation polycondensation: -M-OR + HO-M- → -M-O-M- + ROH(8)

The sol-gel method is practical and commonly used to fabricate chemical sensors for the detection of gas other than NH_3_. The advantages of using the sol-gel method have been thoroughly discussed by Khorramshahi et al. (2018). The sol-gel method is said to be an inexpensive and low-temperature method, which is very suitable for the preparation of zinc oxide (ZnO) nanoparticles. The simplicity, large substrate coating area, and easy control of the film thickness also contribute to the convenience properties of the sol-gel method [111]. In the same year, rGO/CoTiO_3_ nanocomposites were developed using a similar method [112]. The resulting sensor demonstrated an excellent response towards ethanol vapour with a response and recovery time of 2 and 5 s, respectively. A year later, graphene/titanium dioxide (TiO_2_) nanoparticles were also fabricated using the sol-gel method to detect NO_2_ gas at RT with the help of ultraviolet (UV) light [113]. The TiO_2_ was prepared to obtain a colloidal suspension through the hydrolysis and polymerisation of a metal–organic precursor. Tung et al. (2019) also employed the sol-gel method to synthesise rGO in which the poly(ionic liquid) promoted the effective stabilisation and capping agent to control the nucleation growth and prevented the excessive agglomeration of nanoparticles during the synthesis [114]. In their research, the rGO-Fe_3_O_4_ nanoparticles formed simultaneously via the sol-gel approach (in situ technique) or blending of the pre-synthesised Fe_3_O_4_ nanoparticles with an rGO matrix. In the following year, Wang et al. (2020) synthesised black-TiO_2_ (B-TiO_2_) carbon composite powders and fabricated thin film using the sol-gel technique [115]. The prepared B-TiO_2_ demonstrated an enhanced photocatalytic activity and gas sensing performance. Table 4 presents a summary of graphene-based materials synthesised using the sol-gel method as reported in previous studies.

### 4.2. Hydrothermal or Solvothermal Method

The hydrothermal or solvothermal method is usually performed in the presence of polar solvents, such as water, ethanol, methanol, and acetic acid. The synthesis reaction can be conducted at a temperature range of 100–200 °C and a pressure range of ~1 atmosphere. The technique can go on below the supercritical temperature of the water, which is 374 °C. This method is suitable for the fabrication of a composites with specific morphologies and controlled hybrid nanostructure size. The synthesis route is also environmentally friendly because the reaction is carried out at low temperatures using a Teflon-lined stainless-steel autoclave to stimulate the reaction process with very minimal energy consumption. The advantages of using this technique include a greatly increased chemical reaction kinetics with minimal temperature change, the formation of new metastable materials, highly pure final products even from impure feedstock, eco-friendly, and able to prepare hybrid hydroxylated clays and zeolite, which cannot be prepared using other synthesis methods.

The hydrothermal process is a well-known method, especially in synthesising graphene-based composite materials. The ZnO nanoparticles that loaded onto 3D rGO (ZnO/3D-rGO) for carbon monoxide sensing, which were synthesised using the hydrothermal method, possessed a unique porous structure that exhibited the inherent properties of rGO flakes [117]. The as-synthesised ZnO/3D-rGO has an impressive response and recovery time upon detection of carbon monoxide at 200 °C and RT, which is less than 30s due to the high surface area and porosity. The palladium-doped tin oxide/porous rGO (Pd-doped SnO_2_/prGO), which was also fabricated using the hydrothermal method, showed an incredible methane detection at RT with a response and recovery time of 5 and 7 min, respectively [104]. The authors of Nasresfahani et al. (2017) have emphasised the advantages of using the hydrothermal method in fabricating Pd-doped SnO_2_/partially rGO and Pd-doped SnO_2_/rGO thin film sensors.

Additionally, the hydrothermal method is considered as the most ordinary and simplest method for the combination of metal oxides on the graphene nanosheets. The one-pot hydrothermal method contributes to the generation of high crystallized nanostructures without post-synthetic annealing or calcination [118,119]. In addition, this method can retriever the conjugated structure by alteration of the post-reduction defects. It is also a promising technique for the preparation of monodispersed and homogeneous nanoparticles. Kooti et al. (2019) focused on chemiresistor sensors based on SnO_2_ nanorods–nanoporous graphene (NPG) synthesised using a similar method to detect CH_4_ for a detection limit as low as 1000 ppm at relatively low temperature (100–200 °C) [120]. On the other hand, Liu et al. (2017) developed a flower-like rGO-In_2_O_3_ composite using the hydrothermal method for nitrogen dioxide (NO_2_) gas detection [121]. The sensor based on 5 wt% rGO-In_2_O_3_ can operate at RT, with a staggering sensor response of up to 1038%. Meanwhile, the use of a 3 wt% rGO-In_2_O_3_ composite sensor showed a better NO_2_ detection at an operating temperature of 74 °C and a sensor response of 1337%. In the same year, Ye et al. (2017) introduced the rGO-TiO_2_ hybrid material that was synthesised via the same method for NH_3_ detection at RT [122]. The synthesised rGO-TiO_2_ hybrid material showed excellent sensing properties due to the porosity surface of the graphene, with a sensor response of 75%.

Furthermore, Zhao et al. (2018) synthesised SnO_2_-rGO hierarchical porous nanosheets via the hydrothermal route for the detection of ethanol. Hydrothermal or solvothermal is considered the most reliable and controllable route to achieve the preconceived nanostructures. This method introduces GO flakes with a large surface area and abundant functional groups that provide a huge number of attachment sites on the GO surfaces and enable the easy nucleation of SnO_2_ [123]. The SnO_2_-rGO nanosheets have shown a remarkable sensor response, response time, and recovery time of 77.1%, 9 s, and 457 s, respectively, at an operating temperature of 250 °C. Later in the same year, Wang et al. (2018) developed 2D rGO/WS_2_ heterojunction nanostructures for constructing NH_3_ gas sensor. Based on their research, a one-step hydrothermal method was used to effectively synthesise the material, which could perform at RT with an excellent selectivity and stability response to 10 ppm NH_3_ [124] Meanwhile, Sakthivel et al. (2019) developed an rGO-CuO composite with a hierarchical structure for NH_3_ gas detection via the simple surfactant-free method. The developed sensor exhibited a fast response and recovery time of 12 and 90 s [125], respectively. Moreover, Hung et al. (2020) introduced a versatile and scalable synthesis of rGO/WO_3_ nanocomposites via the hydrothermal route for NH_3_ gas sensing application. The study revealed that the capability of the sensor to detect 100 ppm of NH_3_ gas within 37 s and recover within 711 s at 300 °C [126], as listed in Table 5. In the following year, Tohidi et al. (2020) developed a 3D rGO/PANI hybrid material to detect 50 ppm of NH_3_ gas at RT. It was stated that the rGO sheets conjoined to another through π-π stacking and hydrogen bonding creating a 3D scaffold during the hydrothermal process. The PANI nanowires interacted with and attached to some of the vicinity of GO sheets via a similar interaction forming a vast interconnected porous network and, thus, were beneficial to the diffusion of the gas molecules into the hybrid material [127].

### 4.3. Layer-by-Layer (LbL) Deposition Method

**Figure 7:**
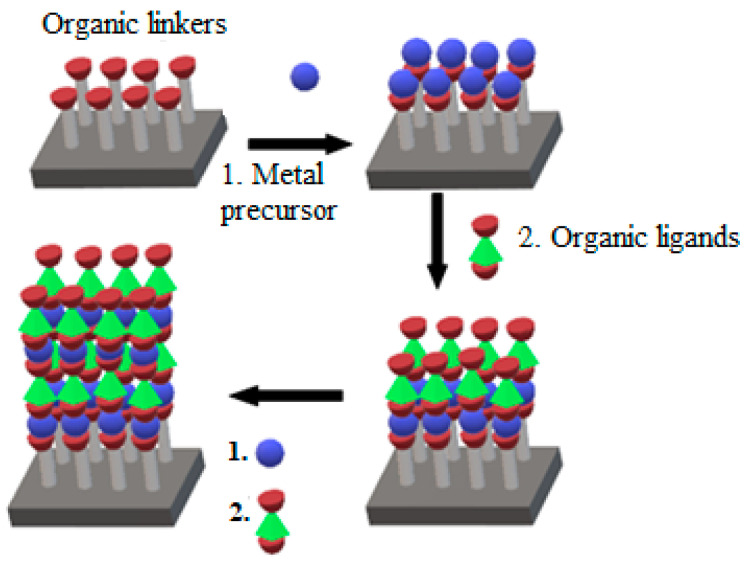
Schematic representation of the LbL deposition method.

The Layer-by-Layer (LbL) deposition method is a highly versatile, simple, cost-effective, and efficient fabrication method of controlled layered structures from numerous component materials. Originally, this method was developed for planar structures and substrates. However, recent studies have used this approach for the development of spherical nanoplates, which produce an LbL assembly freely suspended in water [132]. The synthesis technique normally relates to the surface potential and counterions interface, which allows for the fabrication of organic–inorganic hybrid materials using different surface potentials of organic and inorganic electrolytes. Following the deposition of the organic species in which the initial charge on the surface is countervailed, and a clump of particles is repeatedly sublimated as a layer of negatively charged inorganic building blocks. Multilayer hybrid heterostructure materials with a controlled layer thickness, composition, and function are easily fabricated using this technique due to the fact of its simple step-by-step procedures. Additionally, this technique is useful for the surface functionalisation of nanoparticles. Figure 7 shows the schematic representation of the LbL method for the fabrication of graphene–polymer nanocomposite sensors.

The LbL deposition method has various merits as a surface modification technique for polymer fabrication such as rapidity, environmentally friendly, and economical process. Furthermore, the surface functionality can be directly tuned by selecting the proper polymer or polyelectrolytes, such as poly-(ethylene terephthalate) (PET), poly (allylamine hydrochloride) (PAH), poly(sodium styrenesulfonate) (PSS), polyethene glycol) (PEG), and poly(diallyl dimethylammonium chloride) (PDDA), as shown in Table 6. However, researchers have rarely applied this method in gas sensing due to the presence of critical unresolved issues, specifically in terms of time consumption, and various biocompatibility issues.

### 4.4. Template-Assisted Method

**Figure 8:**
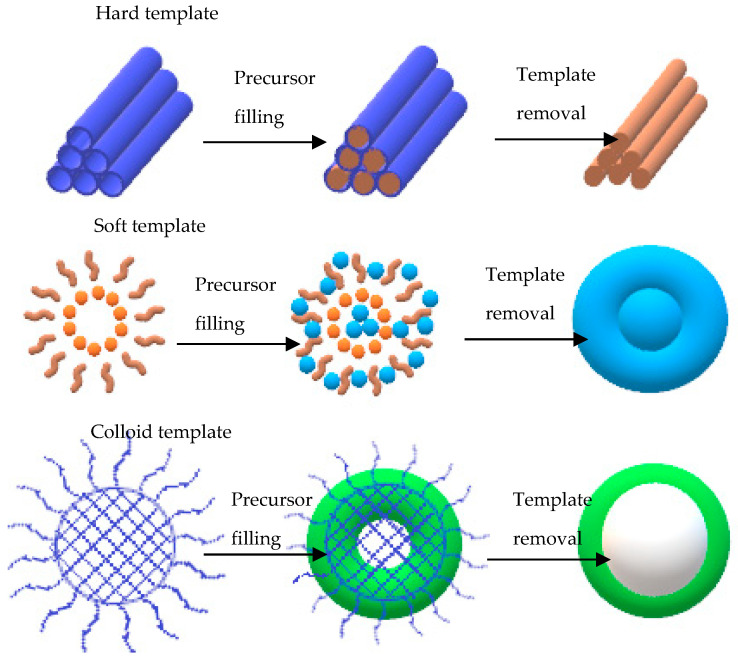
Schematic representation of the synthesised materials using different types of templates. Adapted with permission from [139]. Copyright Elsevier 2021.

Template-assisted synthesis method is an advanced technique for the fabrication of highly crystalline mesoporous and hybrid materials. It can also be used for the fabrication of hybrid nanomaterials with different dimensions, such as 2D or 3D structures. Common 3D structures that can be obtained from this method include hexagonal rod-like structures, lamellar structures, or honeycomb interconnected networks. This method can be further classified as hard-template, soft-template, and colloidal templates, which is the recent approach [132,138]. Basically, the soft template is used for organic-based surfactants, block polymers, or versatile organic molecules. Meanwhile, the hard template is used for inorganic-based ones with silica as the main part of the synthesis. In order to achieve the desired hybrid nanostructures, several criteria need to be considered, such as surfactants concentration, temperature, and pH value. The hybrid nanomaterials can be easily removed from the templates once they are formed, followed by high temperature above 450 °C, which makes the materials highly inorganic. Figure 8 shows a schematic representation of the template-assisted method for the fabrication of graphene–polymer nanocomposite sensors.

**Figure 9:**
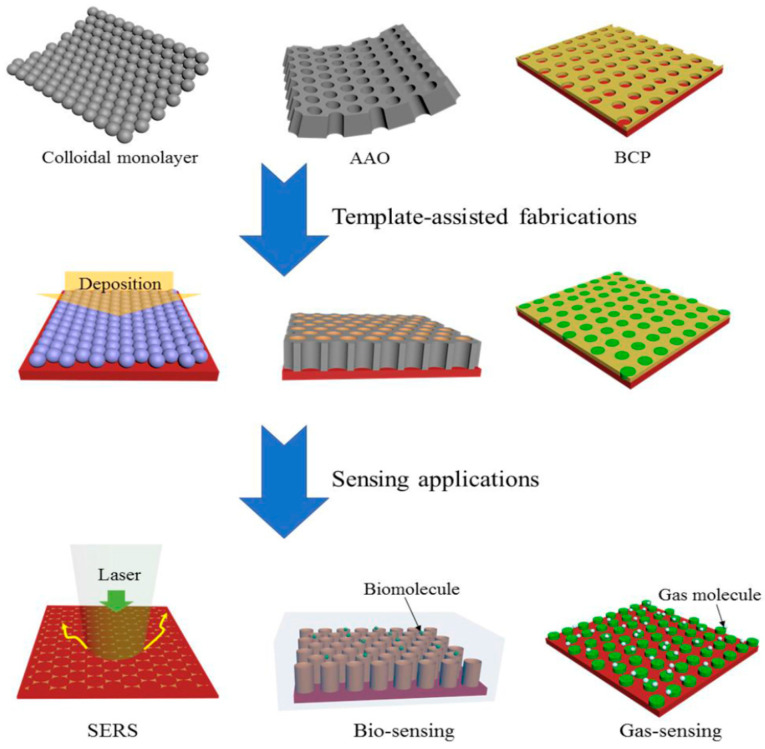
Template-assisted arrays. Adapted with permission from [145]. Copyright Wiley 2018.

To date, several templates have been widely used, including colloidal monolayer, Anodic Aluminium Oxide (AAO), Block Copolymer (BCP), and monoprint mould, as shown in Figure 9. The use of such templates leads to a large surface area and highly ordered array with precise morphological constraints. For instance, the colloidal monolayer is a conventional template that generates an ordered array of nanodiscs, nanotips, or nanopillars. Nanoring arrays can be obtained by wetting around the nanospheres. This template is widely used in gas sensing, photodetector, and Surface-Enhanced Raman Spectroscopy (SERS). In addition, the AAO template is notable for its chemical stability with a wide range of desirable materials. These arrays can also be acquired via the conventional sol-gel technique. Similarly, the CVD method can be used to develop a homogeneous coating with the chosen material on the AAO template, producing a nanotube or a nanopore array. Interestingly, these arrays are reported to be a fairly good sensing platform [140,141,142]. Apart from those templates, BCP and nanoimprint lithography method can be used to prepare an ordered nanostructure array [143,144]. The BCP self-assembles on the substrate and produces orderly pits. The desired materials or reactive ions are then deposited for etching. Consequently, the ordered nanostructure array with remarkable density alignment features can be obtained. On the other hand, the nanoimprint lithography mechanism is more focused on the mechanical deformation of a desired resist. The sample are pressed neatly with a mould. Then, nano- and micropatterns on numerous substrates can be obtained after detaching the mould from the sample [145].

Zou and his co-workers have used soft template synthesis based on the BCP strategy, which is considered the most efficient and flexible method to develop ordered mesoporous materials through the controllable interfacial-induced co-assembly process. The newly designed amphiphilic BCPs consist of high sp^2^ hybridised content of carbon containing BCPs in the hydrophobic segments. This newly designed copolymer is relatively stable, and it practically converts in situ into residual carbon to support the mesoporous structure through free radical polymerisation. This novel strategy forms the sp^2^-hybridised carbon-containing BCPs, such as the ligand-assisted assembly and resolve-assisted assembly strategies, to support the structure of mesoporous metal oxide under extreme calcination temperatures of greater than 400 ℃ and to achieve a controllable and multipurpose mesoporous semiconducting metal oxide synthesis with excellent gas sensing performance [146]. The pluronic-type poly(ethylene oxide)-b-poly(propylene oxide) copolymer (P123 and F127) was employed in the study as a structure-directing agent with the ability to generate a small mesoporous size (<10 nm) and semicrystalline framework owing to the short-chained polymer and poor thermal stability of the template molecules.

In the same year, Zhao and his co-workers introduced the mesoporous WO_3_@graphene aerogel nanocomposites for the detection of low-temperature acetone. The graphene aerogel was used as the mesoporous substrate with a uniformly coated mesoporous WO_3_ on both sides of the graphene sheets through the self-assembly solvent evaporation-induced strategy using di-BCP poly(ethylene oxide)-b-polystyrene as the template [147]. The fabricated WO_3_@graphene aerogel nanocomposites possessed a large pore volume that resulted in high sensitivity with a good response (13 s) and recovery (12 s) to acetone at low temperatures of 150 °C. 

The choice of fabrication method for graphene and polymer material is crucial to fabricate efficient gas sensing platforms with a high density, high surface-to-volume ratio, and convex-rich morphology. However, the limitation of the above-mentioned method is that the fabricated materials tend to form internal structural stress defects and deterioration, which could destabilise the sensor. Although this method has a broad-sized distribution, the morphology parameter of the nanostructure-array-based sensing platform is adjustable.

### 4.5. Physical Vapour Deposition (PVD)

The PVD method is an atomistic deposition process in which the material is vapourised from a solid or liquid source in the form of atoms or molecules and transported through a vacuum or low-pressure gases or plasma environment in the form of vapour to the substrate where it is condensed [148]. Commonly, the PVD technique is used to deposit films with thicknesses that vary from a few nanometres to thousands of nanometres. Further, they can be used to form multilayer coatings, graded composition and extremely thick deposits, and discrete structures [148,149]. Furthermore, the PVD technique can be applied to deposit hybrid nanomaterial films via vacuum deposition or evaporation, sputter deposition, arc vapour deposition, and ion plating. Generally, the sputtering method involves the bombardment of the material by ions to prepare hybrid nanomaterials [30]. A low-pressure gas and a high energy field are used to carry out the ionisation, which creates ions abundance and free electrons. The ions from the plasma are captivated to the target and cause them to deposit on the hybrid composite material. When the ions strike the target, they knock the target atoms loose and coat them on the substrate. However, this method is not suitable and is expensive for large-scale production due to the fact of its inconsistent usage under vacuum conditions.

Previously, PVD has been used to deposit interdigitated Au electrodes with 400 µm interdigitated spacing, 100 nm thickness, and 100 µm width on a flexible substrate. The S and N co-doped graphene quantum dots/polyaniline (PANI) hybrids were developed by Gavgani and coworkers by loading flexible polyethene terephthalate thin film through the chemical oxidative polymerisation. The gas sensor’s performance towards 100 ppm of NH_3_ gas showed an excellent response (~42%) at RT, a fast response and recovery time (115 and 44 s), good selectivity, low cost, flexibility, and wearable characteristics [150]. In the following year, a poly(3,4-ethylenedioxythiophene)-poly(styrenesulfonate) GO (PEDOT: PSS:GO) film NH_3_ gas sensor was introduced by Hasani and coworkers. The gas sensor was highly environmentally friendly, easy to fabricate, and very cost effective, as it used a low-cost solution processing method. The ohmic back contact to n-GaAs (1 cm^2^) was made of a Au-Ge alloy through PVD on the reverse side of the GaAs wafers. Furthermore, the GaAs appeared much cleaner prior to the PEDOT: PSS:GO deposition using a piranha solution. As a result, the NH_3_ gas sensor showed high potential as an active sensing material that fulfilled the industrial need for high speed, high sensitivity, and excellent selectivity. The as-synthesised PEDOT:PSS:GO films demonstrated a high detection sensitivity (194) with a high response and recovery time (95 and 121 s) towards 20 ppm of NH_3_ gas at RT [151]. Tbar et al. (2019) also used a similar technique to fabricate a 3D nitrogen-doped graphene-based framework/PANI (NiNP_3_@3D-(N)GFs/PANI) hybrid flexible gas sensor. The sensing material was fabricated using a simple in situ oxidative polymerisation process. Based on the results, the NiNP3@3D-(N)GFs/PANI) hybrid gas sensor demonstrated a high selectivity (750.2) towards 1000 ppm of NH_3_ with a good response and recovery time (95 and 25 s) at RT [152]. Table 7 illustrates the recent advances on graphene-based materials synthesised using the PVD method.

## 5. Working Principle of Various NH_3_ Sensors

Various detection principles are commonly used for the determination of as ammonia gas including chemiresistive, Quartz Crystal Microbalance (QCM), and Field-Effect Transistor (FET). Specifically, the detection method can be divided into variations, namely, the solid-state sensing method, optical method, and other methods. The solid-state sensing method involves the use of metal oxide-based and conducting polymer sensors. On the contrary, the sole example of the optical method is tunable diode laser spectroscopy, while other less prevalent methods include the electrochemical, surface acoustic waves, and FET sensors. This review highlights three significant gas sensing designs, which include the chemiresistive, QCM, and FET gas sensors. A summary of the recent works on graphene-based polymer nanocomposite gas sensors is presented in Table 8.

### 5.1. Chemiresistive Gas

The chemiresistive gas sensor is one of the prominent types of sensors that can detect harmful gases, such as carbon monoxide (CO), NO_2_, hydrogen sulphide (H_2_S), and NH_3_. The gas sensor comprises sensing material, interdigitated electrodes, and electrical resistance. An active layer is placed over an array of electrodes to evaluate the change in electrical resistance in the presence of the target analytes. Subsequently, the chemical information is translated by the change in two-point contact electrical resistance, which is simply an electrical signal that is examined and need minimum supportive electronics to construct a mobile, compact, and free-standing system. The resulting sensors use a full composition and structure based on the charge transport and adsorption to obtain a good sensor performance. The performance of a chemiresistive gas sensor depends on many vital factors, such as response, selectivity, sensitivity, response time, repeatability, operating temperature, and LOD.

**Figure 10:**
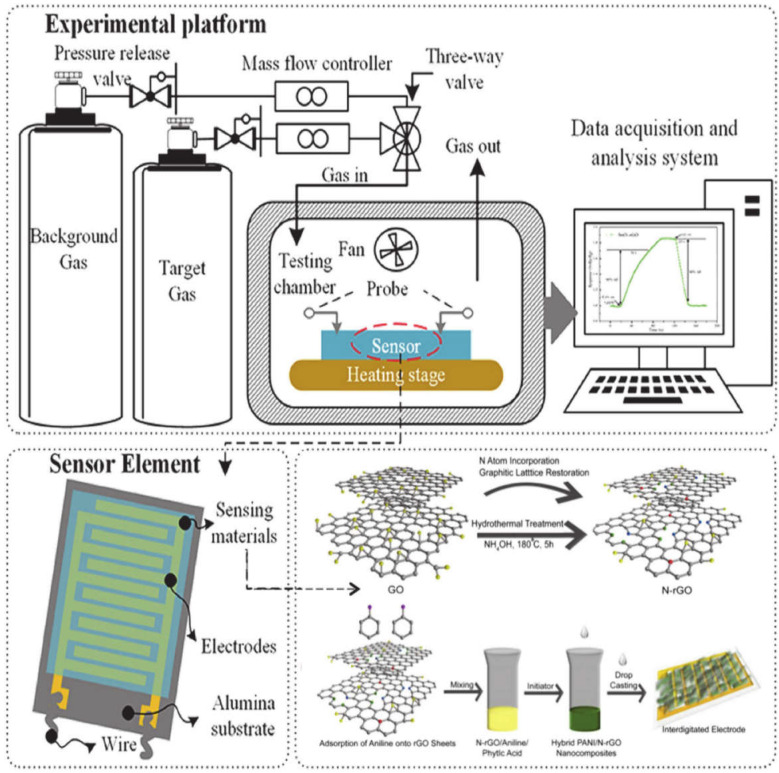
Schematic illustration of a chemiresistive gas sensor showing the electrodes and sensor devices. Adapted with permission from [82]. Copyright MDPI 2020.

Chemiresistors are usually fabricated by coating an interdigitated electrode with a thin film or other sensing material that acts as a bridge between the single gap of two electrodes. Common electrodes used in gas sensors include conductive metals, such as gold and chromium. The interdigitated electrodes acts as a greater substrate surface area that directly interact with the electrode, ultimately increasing the electrical connections and enhancing the overall system conductivity. Additionally, interdigitated electrodes have finger spacing and sizes on the order of microns that can only be arranged via photolithography. The interdigitated electrode and single-gap systems can be aligned in parallel to detect several analytes using a single device. Recent upgrading trials of the existing gas sensor technology have taken place due to the high demand for a wireless, portable, low-cost, and low-power consumption gas sensor, such as low-power Light-Emitting Diodes (LEDs), noble-metal functionalisation, and hybrid materials [8,151,156,157], as seen in Figure 10.

### 5.2. Quartz Crystal Microbalance (QCM)

**Figure 11:**
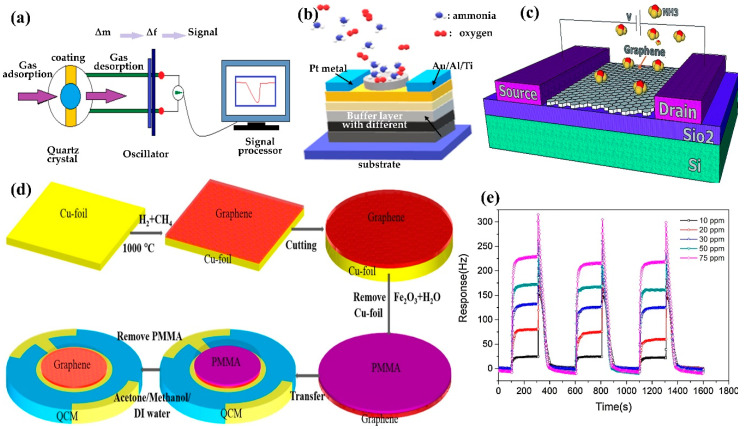
Schematic diagram of the (**a**) QCM, (**b**) tunnel FET, and (**c**) heterostructure electrostrictive FET for NH_3_ detection. Adapted with permission from [169]. Copyright RCS 2016. (**e**) Dynamic response–recovery curves of the QCM gas sensor prepared using GO/chitosan nanocomposite. Adapted with permission from [170]. Copyright Elsevier 2017.

QCM sensors are the most employed mass-sensitive sensors. Typically, the mass of the sensing coating of the QCM changes after the target gases are absorbed. Then, the computer or the signal receiver transforms the mass change (∆m) into the frequency change (∆f) using the Sauerbrey equation (Equation (9)) to depict the sensing performance, as shown in Figure 11a. The QCM sensor effectuates a decreasing frequency trend after the target gases are absorbed on the surface of the QCM. Subsequently, the frequency of the sensor reverts to the initial point immediately after the adsorbed gas desorbs from the surface. The absorptivity of the targeted gas and the sensitivity of the QCM gas sensors can be enhanced by functionalised porous sensing materials, such as functionalised mesoporous silica materials [158], porous metal oxides [159], polymer nanocomposites [160], graphene [161], and metal organics [162,163]. The Sauerbrey equation is defined as follows:

(9) $$∆f=\frac{{2f}_{0}^{2}}{A\surd \rho_{q}{µ}_{q}}∆m$$

where f_0_, ∆f, ∆m, A, ρ_q_, and µ_q_ represent the resonant frequency of the fundamental mode (Hz), normalised frequency change, mass change (g), the area between the electrodes (cm^2^), the density of quartz (ρ_q_ = 2.648 g/cm^3^), and shear modulus of quartz (µ_q_ = 2.947 × 10^11^ g/cm·s), respectively. The equation is derived by assuming the deposited mass as an extension of the thickness of the underlying quartz. Based on Sauerbrey’s equation, the mass to frequency correlation does not depend on the electrode geometry, which allows the mass determination without calibration, thus offering an affordable and time-efficient approach. Figure 11a illustrates the QCM design, Figure 11b,c show the setup and heterostructure of filed effect sensors while Figure 11d shows the QCM-based graphene–polymer nanocomposite sensor. The dynamic response–recovery curves of the QCM gas sensor prepared using GO/chitosan are illustrated in Figure 11e.

### 5.3. Field-Effect Transistor (FET)

FET gas sensors have primarily been developed due to their inherent superior features, including effective operation under harsh and corrosive environmental conditions, ultralow power consumption, high-speed operation, and integrated wireless systems. An FET gas sensor displays vivid changes before and after exposure to the gas analyte, demonstrating the suitability of the 2D FET material for sensitive gas sensing applications. Various types of FET gas sensors have been established, such as tunnel, heterostructure electrostrictive, organic, and polymer FET gas sensors. In addition, an organic FET exhibits check-in electric characteristics, such as the threshold voltage, saturation current, and field-effect charge carrier mobility when the sensors are exposed to the targeted gas. Apart from being lightweight and flexible, FET gas sensors possess numerous advantages, such as excellent selectivity, remarkable repeatability and response, and low-cost production [164,165,166].

Tunnel FETs consist of a conventional Complementary Metal-Oxide Semiconductor (CMOS) that enables supply voltage (V) scaling in ultralow-power and energy-efficient competition. A strong interaction between the device level and circuit level with some modifications of the CMOS circuits is the main requirement of a tunnel FET-based circuit design, to obtain the desired functionalities with optimal energy efficiency. Over the past decades, tunnel FETs have been used to experimentally demonstrate the current and the steep slope in sensor devices. The recent advances in tunnel FETs enable Band-To-Band Tunnelling (BTBT) at the source-channel junction, where the carriers at the high-energy tail of the Fermi–Dirac distribution are filtered by a tunnelling window [167].

Nevertheless, tunnel FET designs face many challenges, specifically the state current, which is restricted by the tunnelling probability and the steep subthreshold slope. Both parameters can be degraded by the thermal energy to determine the Trap-Assist Tunnelling (TAT). The design of a tunnel FET requires the material selection of the system to reduce the tunnelling barrier, achieve good gate electrostatic for the steep and current-on-to-current-off (I_on_/I_off_) ratio, and reduce the interface traps to suppress the TAT. A low band gap can contribute to both the low effective mass and freedom to obtain hetero-band-alignment, which assists in improving the tunnelling probability at low voltage levels. The TAT reduction and steep are caused by the tunnel junctions with a steep doping profile and low defects. Additionally, the gate electrostatics with steep and high current on (I_on_) can be improved by enhancing the gate-control, which converts from the planar device structure into the gate-all-around nanowire structure. The most crucial factor to obtain effective gate control in transistor operation is to determine the quality of the gate dielectrics.

The theoretical framework for a heterostructure electrostrictive FET was discovered and reported by Hemert and coworkers in 2013 [168], which operates based on the principle of voltage-induced strain transduction. An electrostrictive or a piezoelectric material is channelled as a gate oxide layer and inflates when exposed to an applied gate bias. The conversion of out-of-plane stress onto the adjacent channel material has caused stress. It is then followed by the changes of the electronic band structure of the semiconducting channel material of either the bulk silica or 2D material. Thus, the channel could be modified to produce the necessary ON/OFF switching for the FET device’s operation. 

The polymer FET is similar to organic FET which also has been extensively utilized to detect toxic gases, such as NH_3_, NO_2_, H_2_S, alcohols, and others. Organic FET sensors are usually developed using small semiconductors with unique crystalline packing in the thin film, which is responsible for its high charge carrier mobility. However, the fabrication of small molecular semiconducting polymers in a high-vacuum setup is very costly and time consuming. Despite these drawbacks, semiconducting polymers are more effective, easy to process, and compatible with plastic substrates, which make them very advantageous for electronic nose applications [171].

There are several relevant parameters to describe the performance of sensor devices, including sensitivity, selectivity, LOD, stability, and recovery and response time. Sensitivity refers to the ability of the sensor device to detect the minimum concentration of target gases, while selectivity is the capability of the sensor device to distinguish a particular gas from a gas mixture. In addition, the LOD of gas sensors signifies the minimum amount of gas the sensor can detect. The stability of the sensor determines its durability under severe operating conditions, such as high temperature, high pressure, and corrosive environments. Eventually, the recovery and response time refer to the adsorption and desorption speeds of the sensor with respect to the detected analyte, respectively. In other words, both the recovery and response time indicate the amount of time required to reach 90% of the final equilibrium value after the detected gas was injected and removed, respectively. Table 8 provides a summary of recent works related to graphene-based polymer nanocomposite gas sensors.

**Table 8:** Summary of recent works related to graphene-based polymer nanocomposite gas sensors.

Materials	Synthesis Method	Detection Range	Operating Temperature (°C)	Performance				
				Response (%)	Gas Concentration	Response Time	Recovery Time	Reference
rGO/CuFe_2_O_4_ nanocomposites	Modified Hummers’ method and combustion method	50 ppm	RT	9.8 ^a^	50 ppm	3 s	3 s	[172]
Pd/SnO_2_/rGO ternary composite	One-pot synthesis under ultrasonication	5–300 ppm	RT	7.6 ^b^	5 ppm	7 min	50 min	[173]
rGO/bromophenol blue	UV lithography	5–40 ppm	RT	2.6 ^a^	5 ppm	3.5–20 min	1.5 h	[174]
Graphene/PANI/PET film	In situ chemical oxidative polymerisation	10–100 ppm	RT	344.2 ^c^	100 ppm	20 s	27 s	[34]
Py-rGO	Chemical reduction	1 ppb–50 ppm	RT	2.4 ^a^	1 ppb	1.4 s	<6 min	[44]
ZnO nanowire/rGO	Thermal reduction	500 ppb–5000 ppm	RT	7.2 ^a^	500 ppb	50 s	<200 s	[175]
rGO/Ag nanoparticles	Addition of AgNO_3_ and H_2_PtCl_6_ and NaBH_4_	0.5–15 ppm	RT	6.25 ^a^	1 ppm	5 s	6 s	[176]
rGO/Au nanoparticles	Addition of AgNO_3_ and H_2_PtCl_6_ and NaBH_4_	1.5–13 ppm	RT	2.87 ^a^	1 ppm	13 s	17 s	[176]
rGO/Pt nanoparticles	Addition of AgNO_3_ and H_2_PtCl_6_ and NaBH_4_	0.1–15 ppm	RT	0.5 ^a^	1 ppm	7 s	8 s	[176]
rGO/Ppy nanocomposites	Drop cast in situ oxidative polymerisation	3–500 ppm	RT	0.99 ^a^	3 ppm	405 s	-	[45]
rGO/P3HT composite films	Spray process	10–50 ppm	RT	7.15 ^a^	10 ppm	141 s	488 s	[177]
rGO/P3HT composite films	Spray process	10–50 ppm	RT	12.63 ^a^	50 ppm	92 s	415 s	[177]
TiO_2_/rGO layered film	Stepwise deposition of GO and TiO_2_ layers	10 ppm	RT	0.62 ^c^	10 ppm	0.62 s	-	[178]
Cu-BTC/Ppy-rGO nanocomposites	Hydrothermal process combined with in situ chemical polymerisation	10–150 ppm	RT	12.4 ^a^	50 ppm	13 s	22 s	[46]
α-Fe_2_O_3_/graphene nanocomposites	Hydrothermal treatment and dispersion process	10–50 ppm	250	13.5 ^a^	10 ppm	152 s	10.8 min	[179]
α-Fe_2_O_3_/graphene nanocomposites	Hydrothermal treatment and dispersion process	10–50 ppm	250	26 ^a^	50 ppm	70 s	20.3 min	[179]
rGO/graphene	CVD	0.5–50 ppm	RT	4.7 ^a^	10 ppm	150 s	345 s	[180]
Tannic acid/rGO	Chemical reduction	1310–6560 ppm	RT	12.5 ^a^	1310 ppm	40 s	170 s	[181]
Tannic acid/rGO	Chemical reduction	1310–6560 ppm	RT	12.5 ^a^	6560 ppm	20 s	100 s	[181]
PANI nanofibre/rGO	in situ reduction by oxidative polymerisation of aniline	5–50 ppm	RT	47.6 ^a^	50 ppm	-	-	[182]
GO	Chemical reduction	100–1000 ppm	RT	22.2 ^a^	100 ppm	-	-	[183]
PPy/rGO	Hydrothermal treatment	1–4 ppm	RT	6.1 ^a^	1 ppm	1 min	5 min	[47]
rGO-rosebengal composites	Chemical reduction	400–2800 ppm	RT	10.3 ^a^	400 ppm	100 s	-	[184]
rGO/In_2_O_3_ nanocubes	Hydrothermal treatment	100–1000 ppm	RT	3.5	100 ppm	15 s	38 s	[185]
rGO/WO_3_ nanowires	Hydrothermal treatment	20–500 ppm	300–450	35 ^c^	500 ppm	37 s	711 s	[126]
ZIF8-ZnO/rGO nanocomposites	-	0.5–30 ppm	RT	2.6 ^c^	10 ppm	50 s	25 s	[186]
PANI/3D-RGO	Hydrothermal treatment	5–25 ppm	RT	3.72 ^a^	50 ppm	98 s	288 s	[127]
Single-layer graphene	CVD	100–200 ppm	RT	48 ^a^	100 ppm	5 min	15 min	[187]
Ppy/rGO	Oxidation and reduction method	0.1–9.5 ppm	225–330	1.83 ^a^	0.1 ppm	118 s	122 s	[43]
Microfibre structure coated with GO	Chemical reduction	0.04–0.5%	RT	26.99	0.04%	497 s	192 s	[188]
Microfibre structure coated with GO	Chemical reduction	0.04–0.5%	RT	61.78	0.04%	385 s	288 s	[188]
PANI thin films	In situ oxidative polymerisation	10–100 ppm	RT	63.50 ^a^	100 ppm	63 s	979 s	[189]
PANI-rGO nanocomposites film	Ultrasound oscillator	0.3–130 ppm	RT	0.86 ^a^	15 ppm	8 min	48 min	[156]
Worm-like mesoporous Ppy@rGO heterostructure	In situ polymerisation	0.2–40 ppm	RT	45 ^a^	10 ppm	<200 s	10 min	[190]
Ppy + graphene	Electropolymerisation	1–4 ppm	RT	1.77 ^a^	1 ppm	2 min	5 min	[191]

^a^$S=\frac{\left(R_{gas}-R_{air}\right)}{R_{gas}}\times 100\%$; ^b^ $S=\frac{\left(R_{air}-R_{gas}\right)}{R_{air}}\times 100\%$; ^c^ $S=\frac{R_{gas}}{R_{air}}\times 100\%$. RT = room temperature; ppb = part per billion; ppm = part per million.

## 6. Polymer Electrolytes and the Ion Transport Model

Polymer electrolytes are promising components for developing gas sensor devices, especially in the field of solid state. A polymer electrolyte comprises the dissolution of salts in a polymer matrix with high molecular weight. This solvent-free solid has various desirable characteristics such as lightweight, flexibility, high transparency, high ionic conductivity, large electrochemical windows, and easy processability. Moreover, it carries ionic conduction property, which allows it to be extensively utilised in electrochemical devices, such as rechargeable [192] and solid-state batteries [193], electrochemical sensor [194], supercapacitors [195], fuel cells [196], analogue memory devices [197], electrochromic windows [198], and dye-sensitised solar cell [199]. Moreover, these materials not only exhibit excellent leakage prevention and internal shooting but also do not require the use of corrosive solvents and are free of hazardous gas emissions.

**Figure 12:**
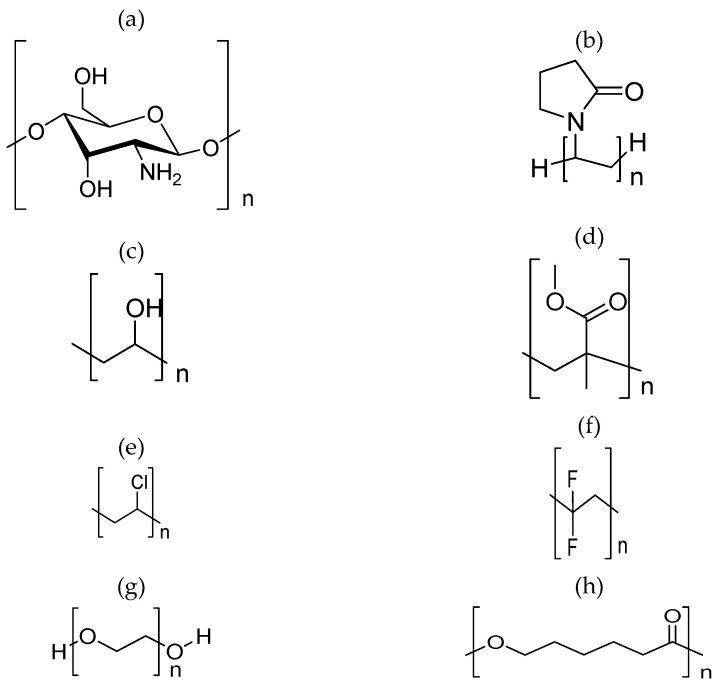
Chemical structure of extensively used polar polymer electrolytes: (**a**) chitosan; (**b**) polyvinylpyrrolidone (PVP); (**c**) polyvinyl alcohol (PVA); (**d**) polymethylmethacrylate (PMMA); (**e**) polyvinyl chloride (PVC); (**f**) polyvinylidene fluoride (PVDF); (**g**) polyethene oxide (PEO); (**h**) polycaprolactone (PCL).

The wide use of polymer electrolytes is also mainly due to the presence of polar functional groups that allow electron donors to form coordination bonds with cations, preventing the formation of a molecular dipole and achieving a low hindrance-to-bond rotation. Polymer electrolytes can be grouped into four different types based on their physical state and composition: (i) dry-Solid Polymer Electrolytes (SPEs), (ii) plasticised polymer electrolytes, (iii) Gel Polymer Electrolytes (GPEs), and (iv) Composite Polymer Electrolytes (CPEs). SPEs have drawn wide interest as an alternative to replacing liquid electrolytes due to the fact of their impressive abilities, such as great mechanical strength, ease of fabrication with desirable shapes, and excellent electrode/electrolyte contact formation. They are synthesised through the dissolution of inorganic salt in a polar polymer matrix. Figure 12 shows the chemical structures of well-known and broadly used polymers as host polymers in polymer electrolytes.

Polymer electrolytes consist of both crystalline and amorphous parts in which the ion transport preferably takes place in the amorphous part. The degree of crystallinity and the viscosity of the polymer electrolytes depends mainly on the ionic conductivity of the polymer electrolytes. The low mobility of the ions in the crystalline area would lead to low ionic conductivity. Meanwhile, polymer electrolytes with low viscosity tend to produce more voids and, thus, lead to high conductivity. Several approaches have been proposed to improve the ionic conductivity of polymer electrolytes, such as polymer blending, incorporation of dopants and ionic liquids, utilisation of comb-branched copolymers, and the addition of inorganic filler, plasticisers, and nanomaterials.

i) Dry-solid Polymer Electrolytes (SPEs)

Research on SPEs was first reported by Wright et al. (1975) [200] with the earliest technological application of SPEs being approved by Amand et al. (1979) [201]. Basically, the concept of SPEs is based on the dissolving of inorganic salts in a polar functional polymer that forms an ion-conducting solid electrolyte. A “dry” SPE made of polyethene oxide (PEO) was investigated in previous reports [202,203] in which neither an organic nor inorganic liquid was utilised during the synthesis of the solvent-free system. SPEs comprise several unique properties, such as solvent-free, high thermal stability, low volatility, leak-proof, and electrical, mechanical, volumetric, and electrochemical stabilities [204,205,206]. SPEs are also notable for their lightweights, high automation process and ionic conductivity, long shelf life, high energy density, flexibility, and ease of processing and fabricating [206,207,208]. Chemically, SPEs can eliminate hazardous gases or corrosive solvent in liquid form from leakage and perform effectively under a wide range of operating temperatures [208,209,210].

Despite its promising properties, the main disadvantages of SPEs include their high crystallinity, which leads to low ionic conductivity, as well as the ambient temperature that contributes to low conductivity and high interfacial resistance [211,212,213]. In order to overcome the poor conductivity of SPEs, plasticisers can be added to SPEs, which may enhance the ambient ionic conductivity by increasing the amorphous area and dissociating ion aggregation and, in turn, improvise the DC electrical conductivity.

ii) Plasticised Polymer Electrolytes

**Figure 13:**
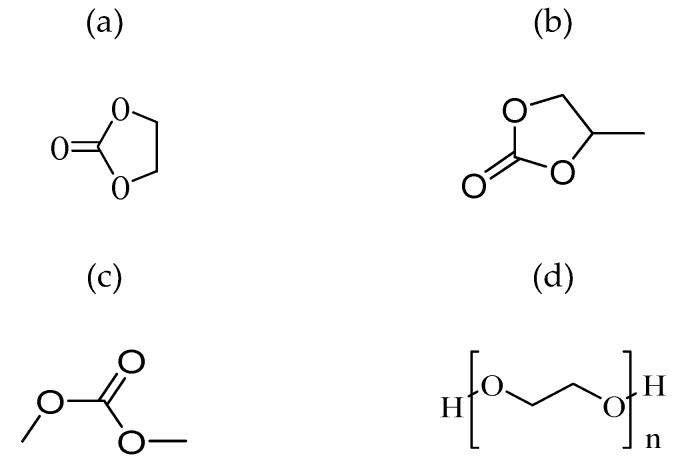
Plasticiser chemical structure of (**a**) ethylene carbonate; (**b**) propylene carbonate; (**c**) dimethyl carbonate; (**d**) polyethene glycol.

Plasticised polymer electrolytes are developed by combining the polymer host with low molecular weight organic compounds, such as ethylene carbonate [214], dimethyl carbonate [215], propylene carbonate [216], and PEG [217]. Plasticisers can enhance the ionic conductivity of the polymer electrolyte by reducing the number of active centres and, therefore, weaken the intermolecular and intramolecular forces between polymer chains. The 3D structure formed on drying turns less rigid and changes the mechanical and thermomechanical properties of the films. The addition of plasticisers allows the reduction of the glass transition temperature of the system thus minimizing the crystallinity of the polymer electrolytes and improving the capability of the salt dissociation with enhanced charge carrier transport. Plasticiser also assists in reducing the semicrystalline phase, which is a nonconducting phase, into the amorphous phase in the matrix [217,218]. Figure 13 shows the plasticiser chemical structures of ethylene, propylene, dimethyl and polyethene. 

One of the main concerns in utilizing these plasticisers is that the resultant electrolyte may demonstrate low mechanical strength [218]. Additionally, adding such plasticisers could raise some concerns over the solvent volatility and reactivity of the polar solvents with lithium electrodes. Therefore, an efficient plasticiser must fulfil these criteria, such as low viscosity, miscibility with polymer host, low volatility, high capability to solvate lithium salts, and electrochemically stability [218]. Hence, the selection of plasticiser is crucial to achieving maximum suitability. In order to overcome the weakness of plasticisers, it is suggested to use natural cellulose microcrystals that can reinforce the mechanical strength and increase the thermal stability [218].

iii) Gel Polymer Electrolytes (GPEs)

A GPE is also known as plasticised polymer electrolyte due to its similar structural properties to plastics. A GPE is a plasticised or gelled polymer matrix that results in the expansion of the polymer matrix in a liquid electrolyte. The GPE is easily produced by heating the polymer matrix mixture comprising PEO and an alkali metal salt, for instance lithium salt and zinc salt, and a large amount of organic solvent or plasticiser. The viscous clear solution is then cast in a hot state and left to cool under the electrode’s pressure to generate a thin film. The polymer acts as a host matrix to trap the liquid constituents. Subsequently, the combination of plasticiser molecules generates a interconnected network in which the ion conduction connected to the host polymer and, thus, provides structural support.

GPEs display high ambient conductivity, poor volatility, safe operation, low reactivity, and great chemical, mechanical, photochemical, electrochemical, and structural stabilities [219,220,221]. The polymer is also notable for its lightweight, flexible, solvent-free, high-energy density, good volumetric stability, vast electrochemical windows, and easy configuration into the desired size and shapes [222,223,224]. The advantages of GPEs enhance their capability in various electrochemical applications, especially in battery production. Furthermore, the addition of GPEs enhanced devices’ safety by preventing leakage and internal shorting, which prolonged the shelf life of the batteries [225].

However, few studies have reported the drawbacks of GPEs, such as the impregnation of liquid electrolytes and volatile solvent residual discharge, which leads to poor mechanical strength and abrupt metal electrode reactivity increment that may hinder the wider application of GPEs [226,227,228]. The poor mechanical strength of GPEs could result in the inability to resist the stress between the anode and cathode. The undesirable qualities can be overcome by adding fillers and nanomaterials. Although the influence of plasticisers on the percolative behaviour of ion transport may lead to poor ionic conductivity, the use of plasticisers with suitable liquid solvents containing a high dielectric constant, ε, and low viscosity, η, would produce GPEs with high ionic conductivity. Eventually, the rapid growth of the amorphous regions following the adsorption of the liquid leads to the percolation threshold at ambient temperature. The developed network in the amorphous region accelerates the ion conducting pathways that increase the ion mobility and, thus, generate higher ionic conductivity.

iv) Composite Polymer Electrolytes (CPEs)

CPEs are developed to overcome the limitations of SPEs mainly the presence of ion pairs in SPEs, called the ion association, and ion triplets, which contribute to the weakening of the dielectric constant of the host polymers [229]. Inorganic inert fillers with high dielectric constant have been introduced in the polymer electrolytes to improve the qualities of SPEs. The dielectric permittivity can be calibrated by carefully choosing the type and the amount of the incorporated inorganic filler material. For example, ceramic materials are familiar inorganic fillers that are essentially fragile and exhibit poor dielectric strength. Although all polymers have considerably low dielectric permittivity, they are flexible, easy to process, and can sustain high fields. Hence, the combination of polymer electrolytes and ceramic materials enhances ion mobility and offers outstanding features, such as excellent interfacial contact, high flexibility and thermal stability, , and high ionic conductivity [230,231].

Various methods have been introduced to fabricate CPEs, for instance, binary salt systems [230], polymer blending [232], incorporation of additives, such as plasticiser [233], crosslinking polymer matrices [234], impregnation with ionic liquid [235], doping of nanomaterials [236], reinforcement by inorganic fillers [230], and comb-branched copolymer [237]. The characteristics of the particles used, including the particle sizes, concentration, surface area, porosity, and the interaction between particles and the polymer matrices, are crucial to determining the electronic and ionic conductivities of the CPEs [238,239,240].

## 7. Organic Conducting Polymer Nanocomposite

The rapid development of effective NH_3_ sensing devices is crucial for human and environmental safety. Conventional detection techniques, such as calorimeter, optical spectroscopy, and chromatography, have been frequently used to detect NH_3_ over the past several years. However, these conventional techniques have failed to overcome certain limitations, such as time-consuming sample pre-treatment and preparation, the requirement of complex instrumentation, and considerably high operating cost. Despite the development of various sensors with excellent sensitivity and selectivity, quick response, and remarkable LOD, they are somehow hindered from applying the conventional detection methods. In that respect, recently developed nanotechnology sensors have enabled the fabrication of novel materials with outstanding sensing performance.

**Figure 14:**
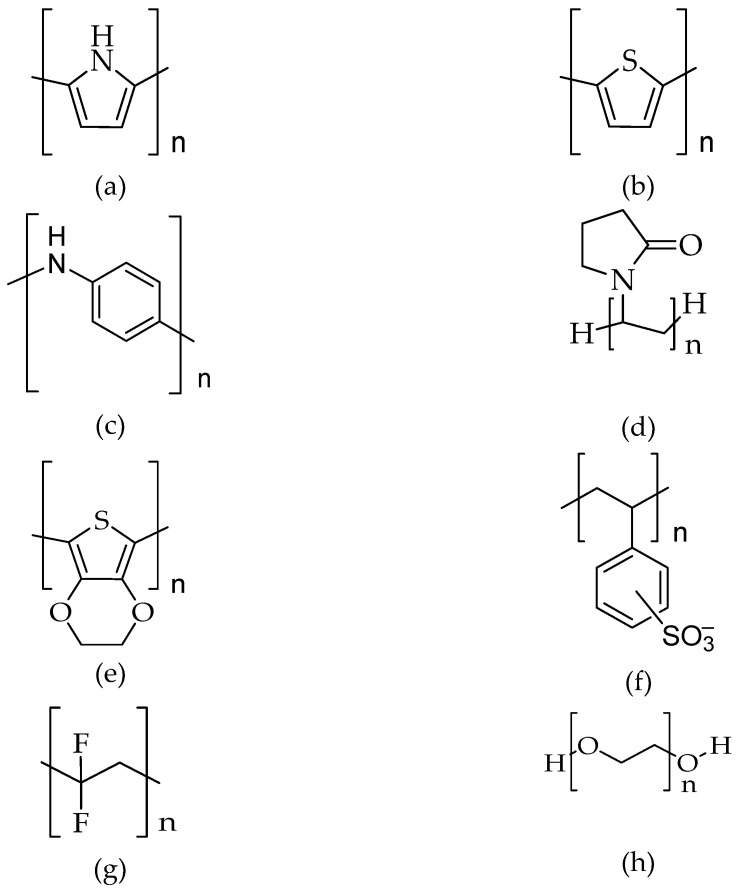
Chemical structure of the polymers used in the development of NH_3_ hybrid nanocomposite sensors: (**a**) polypyrrole (Ppy); (**b**) polythiophene (PTh); (**c**) polyaniline (PANI); (**d**) polyvinylpyrrolidone (PVP); (**e**) poly(3,4-diethylenedioxythiophene (PEDOT); (**f**) poly(styrene sulfonate) (PSS); (**g**) polyvinylidene fluoride (PVDF); (**h**) polyethylene glycol (PEG).

Polymers are promising materials in the development of sensor devices. These large molecules are composed of chains or rings of repeating subunits of monomers and tend to exhibit high melting and boiling points due to the strong bonding of the high molecular-weight molecules. Polymers can be grouped into two types, namely, organic (polymers with carbon as their backbone) and inorganic (polymers with other elements as their backbone). Organic conducting polymers are frequently used in the development of gas sensors due to their ion mobility and conductivity properties. As shown in Figure 14, organic conducting polymers, such as polypyrrole (Ppy) [43], polyaniline (PANI) [155], polythiophene (PTh) [241], poly(3,4-diethylenedioxythiophene) (PEDOT) [242], polyvinylidene fluoride (PVDF) [243], polyvinylpyrrolidone (PVP) [244], PEG [245] and poly(styrene sulfonate) (PSS) [246], are popular materials in the fabrication of NH_3_ gas sensors. PANI, Ppy, and their combination as well as the PEDOT:PSS combinations have received the most attention among researchers given their unique redox properties, low cost, good electrical conductivity, good stability, ease of synthesis, a good response at RT, highly sensitive, and offer a high potential to be applied in many fields, such as corrosion protection [247,248,249], biosensing [250,251,252], photocatalysis [253,254,255], biomedical [256,257,258], supercapacitor [259,260,261], energy storage [262,263,264], and gas sensors [265,266,267]. Conducting polymers have become an area of interest over many years and have been successfully used to develop conducting nanocomposites. Moreover, a hybrid nanocomposite system comprising metal oxides and carbonaceous materials was listed as one of the most promising organic conducting polymers. The combination of the nanostructured particles and conducting polymers was shown to enhance the properties of the polymers and, thus, achieved an incredible sensing performance [156,157,268]. Figure 14 demonstrates the chemical structures of various conducting polymers used for the fabrication of ammonia gas sensors.

Conducting polymers comprises several long chains with extended π-systems permittable to chemical and electrochemical oxidation or reduction. Their electrical and optical properties could be tuned and modified by controlling the oxidation and reduction (redox) process, which are both often reversible. Thus, the electrical and optical properties can be systematically controlled by switching from a highly conductive semiconductor to a less conducting or insulative one with excellent precision. Hence, conducting polymers can form conductive macromolecules with a fully conjugated sequence of bonds and backbones that contain both positive and negative charges via the redox reaction. In recent years, multiple studies have been attempted to incorporate graphene into conducting polymer matrices to develop nanocomposites with novel properties, such as large surface-to-volume ratio, enhanced electrical, mechanical, thermal, and to suit and enable exploration various applications [249,261,267].

The PANI-based gas sensor has received the highest attention compared to the other conducting polymers used for fabricating gas sensors. Different types of nanoparticles in PANI polymeric matrix could produce different responses to ammonia gas, mainly due to the synergistic effect. For instance, PANI is a p-type polymer with excellent properties as an extrinsic semiconductor with a majority of hole carriers and a minority of electron carriers, a large electron concentration compared to the hole concentration, and acceptor energy levels that are very close to the valence band. Generally, a PANI-based sensor consists of different structures, such as films, thin films, nanofibrous thin films, nanowires, nanofibres, and pellets, which have acquired response values below 100% at a gas concentration of 20–1000 ppm [155,156,189]. Tohidi and his co-workers mixed PANI thin films with rGO aerogel to detect NH_3_ gas at RT. The rGO aerogel was synthesised via the hydrothermal method and exhibited high porosity, which is favourable for gas adsorption. The PANI nanofibers interconnect with the fibrillar network, which grows vertically on the surface of the 3D-rGO nanosheets. The high surface area of the synthesised material enhanced the exposure to the NH_3_ gas which, in turn, contributes to the high sensing performance. The PANI/3D-rGO hybrid sensor has a high response of 111% to 5 ppm of NH_3_, with a response and recovery time of 85 and 187 s [155], respectively. Lee and his co-workers indicated that the combination of the rGO nanosheets with PANI improved the sensor response to NH_3_ gas. The rGO provides an electron transfer path to PANI and enhances the electron transfer speed using the π-π conjugated bond reaction of rGO and PANI. Thus, the synthesised PANI-rGO nanocomposites demonstrated an incredible sensing response of 0.86% for the detection of 15 ppm of NH_3_ with 8 and 48 min of response and recovery time [156], respectively.

The Ppy/graphene sensor is one of the most widely utilized conducting polymers for ammonia sensing due to its high conductivity, easy synthesis process, and great environmental stability [269]. The oxidised Ppy is stable under ambient conditions and high temperatures of greater than 300 °C [267]. The oxidation of Ppy has been well studied and characterised due to the fact of its extreme susceptibility to oxidation. The electrochemical properties of Ppy enable the formation of high-quality films. The structure of pure Ppy appears as a cauliflower-like structure. The thickness of the pure Ppy film must be carefully controlled, since an extremely thin film may cause an incomplete cover of the electrode gap between the neighbouring electrodes. In contrast, an exceedingly thick film would reduce the conductivity value. Interestingly, the introduction of rGO enhanced the conductivity of Ppy and lowered the power consumption. At 1 ppm of NH_3_, the Ppy/rGO nanocomposites recorded a sensing response of 6.1%, with a response and recovery time of 1 and 5 min, respectively, at RT [47]. The mobility of the Ppy-based sensor depends on the synthesis method, which is most frequently performed via the in situ polymerisation method [82]. The in-situ polymerisation of monomer on graphene demonstrates an excellent synergistic effect on the conducting polymer and graphene composites. The synergistic effect of the large specific surface area and worm-like mesoporous heterostructure of the Ppy/graphene plays a crucial role in producing a remarkable sensing response, which triggers gas diffusion and carrier transport. Gao and co-workers proved the synergistic effect of the w-Ppy@rGO heterostructures on the detection of 10 ppm of NH_3_, with a considerable response value of 45% at RT [190]. The synthesised Ppy via electropolymerisation and graphene also achieved an outstanding sensing performance with great stability, reproducibility, and tolerance to humidity [191]. Since the Ppy layer acts like a p-type semiconductor, the adsorption of NH_3_ molecule on the Ppy surface induced the bonding interactions between NH_3_ molecules and the Ppy. The electron transfer during the adsorption of NH_3_ molecules led to the reduction of the hole concentration of the Ppy layer, subsequently increasing the Ppy resistance. On the other hand, the graphene acts as a p-type semimetal. Overall, the functionalisation with the Ppy has made it possible to receive the electron transfer from NH_3_ molecules, thus increasing the graphene resistance as well.

The PEDOT:PSS-based conducting polymer sensor is one of the most extensively researched NH_3_ gas sensors due to the fact of its unique features, such as excellent solution-fabrication capability and miscibility, good controllable conductivity, exceptional chemical and electrochemical stability and good biocompatibility and optical transparency [270,271]. The combined conducting polymer and graphene composite improves the electrons mobility of the sensor for NH_3_ gas detection. Previously, the combined graphene quantum dots dopant sensor with PEDOT:PSS showed an excellent response (212.32%) towards 1500 ppm of NH_3_ molecules at RT [270]. The sensor formed a p-n heterojunction in which the detection recorded a resistance change upon exposure to NH_3_ gas. There are greater electron hopping which occurr at the shorter PEDOT chains than in between the longer PSS chains. The graphene quantum dots are conductive pathways and act as a charge carrier that causes swelling and increases the PEDOT distance in the film. In one study, the concentration of carbon-based material embedded in PEDOT: PSS highly affected the type of NH_3_ adsorption that occurred during the adsorption and desorption process [242]. 

All conducting polymers can easily endure redox reactions compared to conventional polymers mainly because conducting polymers possess double bonds of a π-bond and σ-bond. The electrons in σ-bonds usually originate from the backbone chain that forms a long-saturated chain, which provides the conducting polymers with considerable mechanical properties. Meanwhile, the electrons in the π-bonds are delocalised along the chain due to the overlapping of the π-orbital with the neighbouring molecules of the conjugated bonds, which contributes to the conducting and semiconducting properties. The presence of π-conjugated structures in the conducting polymers generates charge carriers in the form of free electrons or holes. In turn, the charge carriers tend to delocalise over the conjugated polymer chain and make it easy for the polymer to form a conjugated backbone [272,273]. In contrast, the charge carriers should be introduced extrinsically by the charge transfer process or known as doping when the conjugated polymers do not have any intrinsic charge carriers. The energy band gap usually decreases following the doping process. For example, the band gap of polyethylene in a fully saturated chain is ~5 eV and decreases to approximately ~1.5 eV in a fully conjugated structure of polyacetylene with the intrinsic conductivities of ~10^−17^ and ~10^−8^ S/cm [274], respectively.

The doping and undoping processes play an important role in the gas sensing mechanism fabricated using conducting polymer and graphene fillers [275]. Doping is the process of adding an impurity (called a dopant) to an intrinsic or pure material, which acts as a charge transfer agent. After doping, the intrinsic material becomes an extrinsic material that can be used in gas sensing. The dopant is used to remove or add electrons to the polymers. Dopants increase the electrical conductivity through p-type doping for the oxidation process or n-type doping for the reduction process. The oxidation level of the conducting polymer affects the sensing response of various analytes. The oxidation level is related to the doping and undoping process, as it is similar to the oxidation and reduction mechanisms between the attracted analytes and the polymer. The process can be observed in the conducting polymers upon exposure to various redox-active gases, such as I_2_, H_2_S, NH_3_, and NO_2_ [156,190,276,277,278]. Electron acceptors, for example O_2_ and NO_2_, can eliminate the electrons from the conjugated benzene rings of the conducting polymers, forming more holes and reducing the electrical resistance. In the case of p-type conducting polymer, the holes are filled up with the electrons from electron-donating gases, such as NH_3_ molecules, which increases the resistance. Nevertheless, the purging of the conducting polymer sensing layer with dry nitrogen or air would partially or fully recover the original resistance.

(10) $$\mathrm{Oxidation}:\;P+{\mathrm{nX}}^{+}A^{-}\to \left[{\mathrm{PnX}}^{+}\right]\left[{\mathrm{nA}}^{-}\right]\;(p-\mathrm{type}\;\mathrm{doping})$$

(11) $$\mathrm{Reduction}\text{:}\;P+{\mathrm{nX}}^{+}A^{-}\to \left[{\mathrm{PnA}}^{-}\right]\left[{\mathrm{nX}}^{+}\right]\;(n-\mathrm{type}\;\mathrm{doping})$$

where P represents the part of the polymer chain. The cation and anion are formed in the first step, then electron is transferred, which leads to the formation of dication and dianion (called the bipolaron). Other polymer segments can interact with the charged segment to provide indefinite lengths of polymer segments. The electrical conductivity increases when reactions between conjugated polymers and oxidants (the p-type doping as an acceptor) or reductants (the n-type doping as a donor) were occurred. There are several known p-type dopants or oxidants, such as FeCl_3_, NH_4_BF_4_, HClO_4_, I_2_, SO_3_CF_3_, AsF_5_, HNO_3_, HCl, H_3_PO_4_, and H_2_SO_4_. On the other hand, commonly known n-type dopants or reductants include Na, Li, and K. The general expression for the doping of a conjugated polymer is as follows:

(12) $${P+2\mathrm{FeCl}}_{3}\to [P^{n}][{\mathrm{nFeCl}}_{4}^{-}]+{\mathrm{FeCl}}_{2}\;(p-\mathrm{type}\;\mathrm{doping})$$

(13) $$P+\mathrm{nNa}\to [P^{n}][{\mathrm{nNa}}^{+}]\;(n-\mathrm{type}\;\mathrm{doping})$$

On the other hand, the undoping or dedoping process which is also known as the compensation or electrical neutralisation of a doped polymer is the process in which p-type doped polymer reacts with several reducing agents and regains its insulating state. The undoping agents diffuse into the polymer matrix and neutralise the charges of the system through a charge-transfer reaction. The undoping process involves chemical reactions between the undoping agents and dopant or carbonium ion, which leads to neutralisation by charge transfer. The undoping process has a high possibility of being affected by thermal treatments. The common undopant agents used for the undoping process include NH_3_, water, and hydrazine. The chemical reaction of the process is shown in Equations (14)–(16):

(14) $${8\mathrm{NH}}_{3}\to {6\mathrm{NH}}_{4}^{+}{+6e}^{-}{+N}_{2}$$

(15) $${6H}_{2}O\to {4H}_{3}O^{+}{+4e}^{-}{+O}_{2}$$

(16) $$\left(\pi -\mathrm{polymer}\right)n^{+}{+\mathrm{ne}}^{-}\to {\left(\pi -\mathrm{polymer}\right)}_{\;}$$

The presence of certain functional groups on the surface of the polymers may affect the sensing signal. The intercoiled and compressed polymeric structure would result in a low response due to the lack of unexposed functional sites, while the strong response from porous or mesoporous structures may due to the large amount of functional group exposure. The involvement of graphene nanomaterials and polymeric systems highly affects the surface area of the hybrid nanocomposites. The electrochemical deposition of the conducting polymers promotes a greater porosity and high surface area. In contrast, the solution-cast films of conducting polymers usually introduce low porosity [279,280,281]. The wrapping of nanomaterials by the polymer through the in situ polymerisation method for the synthesis of conducting polymer nanocomposites under optimal conditions leads to an increment of surface area. When the specific surface area is similar, a greater number of pores are observed to facilitate the gas diffusion and promote the accessibility of the molecules to absorb onto the surface of the nanocomposites, which can be observed through the change in the resistance compared to the nanomaterial with less porosity [155,189,190]. In addition, the change in the humidity can play a role in determining the sensing response of the PEDOT: PSS sensor to NH_3_ gas. 

The temperature and humidity are the two main environmental factors that affect the sensing performance of conducting polymer-based sensors [270,282]. An increment in temperature and humidity leads to the increased conductivity of the conducting polymers, especially for chemiresistive sensors [43,126]. The temperature variable regulates the gas molecules’ adsorption and reaction between the sensing element and analytes. Since most adsorption prefers low-temperature conditions, an increase in the temperature would shift the reaction equilibrium and induce analyte desorption. Subsequently, the adsorption and desorption of analytes can determine the sensitivity of a sensor. Thus, temperature plays a crucial role in determining the sensitivity of a sensor, which affects the reaction rate of the adsorption and desorption of analytes. Meanwhile, the humidity relates to the competition of the adsorption between analytes and water molecules, which affects the sensitivity of the sensor. Both types of molecules become competitive and, consequently, water molecules occupy some of the active sites. The sensitivity of the sensor for the targeted analytes decreases as the humidity level increases [282].

## 8. Detection Mechanism of Ammonia

**Figure 15:**
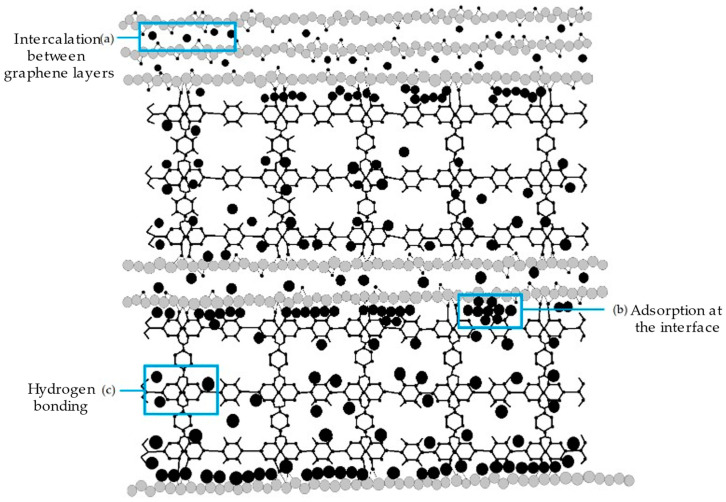
Schematic representation of the adsorption mechanism of NH_3_ (black circle) in the composites, including (**a**) the intercalation between the graphene layers; (**b**) the adsorption at the interface between the graphene layers and the other segments; (**c**) hydrogen bonding with the oxygen atoms of metal oxides in the segments. Adapted with permission from [287]. Copyright ACS 2010.

Graphene exhibits the highest potential in adsorbing NH_3_ due to the fact of its large surface area. NH_3_ can generate the formation of hydrogen bonds between its hydrogen atoms and the oxygen atoms of graphene and its derivatives such as GO, rGO, etc. [283,284,285,286]. NH_3_ induces the collapse in metal oxide structures due to the various similarities between NH_3_ and water in terms of chemistry. Firstly, both inorganic molecules have a strong propensity to form hydrogen bonds with oxygen atoms of any compound in the formation of hybrid nanomaterials. Hydrogen bonds are intermolecular forces that form a unique dipole–dipole interaction when a hydrogen atom is bonded to a strongly electronegative atom that exists in the vicinity of electronegative atoms with a lone pair of electrons. Comparatively, hydrogen bonds appear to be noteworthy among other types of bonding due to the fact of their strong, attractive, and directional characteristics. Hence, the complex hydrogen-bonded networks enhance the adsorption of NH_3_ on amenable surfaces, which is very intriguing. Finally, both molecules have a high potential for future efforts toward a hydrogen-based economy. While water is essential to living things due to the fact of its ubiquity in the chemistry of living systems, NH_3_ is widely used in manufacturing fertilisers, making it the most important industrial chemical production. Both of them, NH_3_ and water molecules, are mainly used for hydrogen production (water–gas shift reaction) or hydrogen storage (NH_3_ as a hydrogen vector). NH_3_ is likely adsorbed on the composites via three mechanisms: (1) the intercalation between the graphene layers from the GO; (2) adsorption at the interface between the nanomaterial segments and the graphene layers, where the dispersive forces are enhanced, specifically the synergic effect of the structure; and (3) hydrogen bonding to the metal oxide tetrahedral via the distortion of the graphene materials structure, as shown in Figure 15 [287].

Numerous gas sensors use conducting polymers to functionalise in the redox reaction for NH_3_ gas sensing. When Ppy interacts with NH_3_ molecules, the doublet of nitrogen in the polymer backbone loses its electron, which is transferred between the positive holes of the Ppy and NH_3_ molecules. This transfer causes the charge-carrier concentration that reduces the conductivity of the sensor. The interaction mechanism between Ppy and NH_3_ is shown in Equation (17):

(17) $${\mathrm{PpyH}}^{+}{+\mathrm{NH}}_{3}\to {\mathrm{Ppy}+\mathrm{NH}}_{4}^{+}$$

The reverse reaction takes place in the air in which ${\mathrm{NH}}_{4}^{+}$ decomposes into NH_3_ and increases the conductivity of Ppy. When Ppy reacts with the electron-donating gases, such as NH_3_, the hole concentration reduces and the resistance increases. New holes are formed within the Ppy structure if and only if the electron-acceptor analytes, such as alcohols, are used to react with Ppy, which leads to decreased electrical resistance [288,289,290,291]. Purging the Ppy layer with dry nitrogen or air can partially or fully replenish its resistance. The ammonia sensing mechanism involves adsorption and desorption, as expressed in Equations (18) and (19):

(18) $${\mathrm{Ppy}}^{+}{+\mathrm{NH}}_{3}\to {\mathrm{Ppy}}^{0}{+\mathrm{NH}}_{3}^{+}\;\;(\mathrm{Adsorption})$$

(19) $${\mathrm{Ppy}}^{0}{+\mathrm{NH}}_{3}^{+}\to {\mathrm{Ppy}}^{+}{+\mathrm{NH}}_{3}\;(\mathrm{Desorption})$$

**Figure 16:**
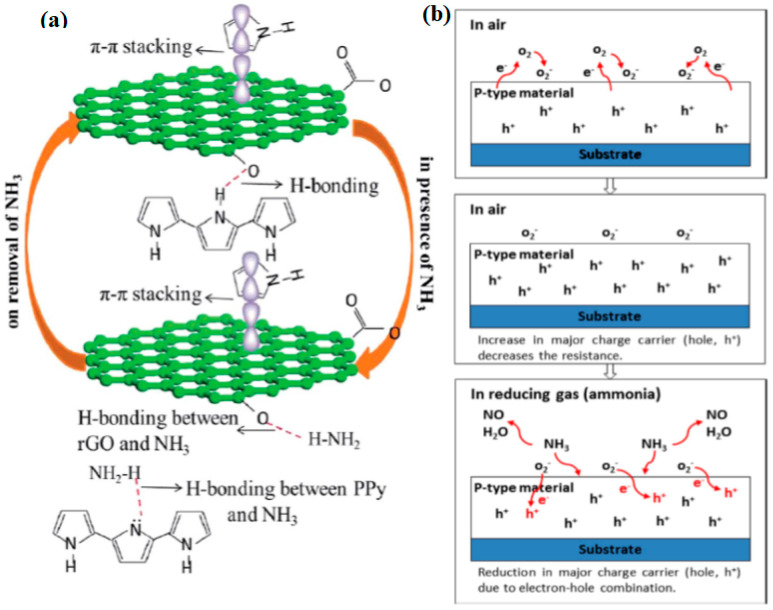
Sensing schematic interaction of (**a**) Ppy/rGO hybrid with NH_3_. Reprinted with permission from [299]. Copyright RSC 2018. (**b**) The sensing mechanism of the p-type material in air and reducing gas environments. Adapted with permission from [275]. Copyright IOPScience 2019.

The mechanism indicates that the p-type Ppy is expected to be undoped under the NH_3_ environment. During the NH_3_ gas sensing, a single pair of electrons or an electron pair from a nitrogen atom is donated to the initial oxidised Ppy, reducing the electrical conductivity, which then forms a neutralised Ppy. There is one possibility of a reversible interaction mechanism occurring between the Ppy and the NH_3_ that may cause a decrement in the doping level of the Ppy through the compensation of the original dopant effect [292,293,294]. The proton transfer between the Ppy and NH_3_ takes place during the desorption of the NH_3_ molecules, which provides an opportunity for the NH_3_ molecules to attack the proton of Ppy and forms ammonium ion [293]. In summary, the doping process is essential to controlling the conductivity of the conducting polymers, thus affecting the gas sensing performance. The formation of hydrogen bonding and π-π stacking between superior combination of Ppy and graphene layers is proven by the excellent sensing performance, which results in faster charge carrier transport and high availability of the active surface site for NH_3_ molecules. As the NH_3_ molecules are absorbed into the sensor, the resistance is reduced due to the release of electrons into the conduction band, which is transferred through the highly mobile graphene [295], as shown in Figure 16.

**Figure 17:**
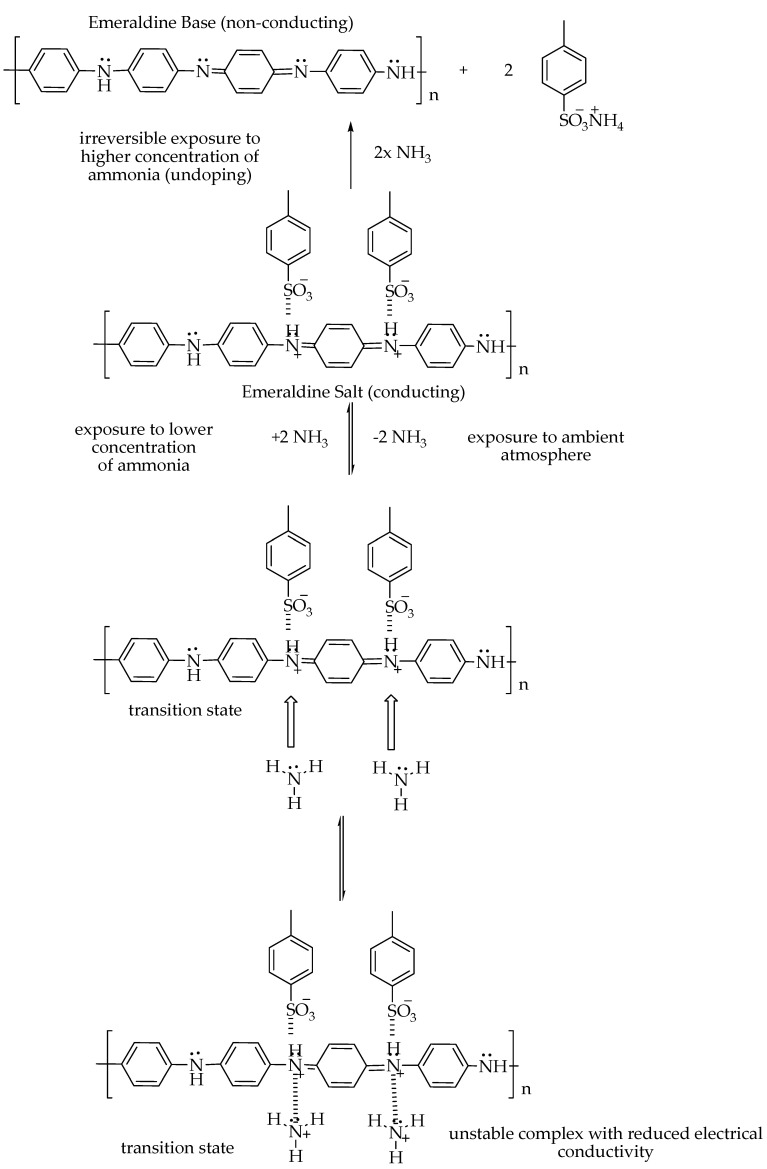
The schematic sensing mechanism of PANI, which involves the chemisorption–desorption phenomenon and electrical compensation.

As for the PANI-based nanocomposite sensing mechanism of NH_3_ molecules, the gas sensing response is similar to the combined adsorption and desorption process and electrical compensation as a side reaction. This process involves chemical bonding and weak forces, such as hydrogen bonding, and van der Waals forces, due to the fact that it is much easier for weak interactions compared to strong interactions [296,297]. The reversible interaction occurs at low concentrations, whereas the irreversible interaction occurs after a short exposure to high concentrations of NH_3_ gas [298]. The interaction of PANI to NH_3_ molecules is similar to that of acid/base reactions in which, at a high concentration of NH_3_, the neutralisation of the dopant acid occurs quickly, which leads to the formation of a nonconducting emeraldine base. Nonetheless, at low concentrations of NH_3_, the chemisorption of NH_3_ molecules takes place on the positively charged nitrogen atoms, as depicted in the schematic mechanism in Figure 17. In addition, the combination of PANI and graphene-based nanomaterials allow the fabrication of high-performance gas sensing platforms due to the increase electroactive surface area of the sensors, rapid electrons movements and high selectivity.

## 9. Future Outlook and Opportunities

Since its first synthesis over the last two decades, graphene is globally known for its high potential for various applications. Graphene has all the reasons to be considered a promising material with high potential in gas sensor-based applications. The introduction of graphene into polymers has further enhanced the potential of these nanocomposites to improve the performance of gas sensors.

The sensing mechanism should be implied and well understood to synthesise exceptional combinations of graphene based polymeric nanocomposites with desired functionalities and morphologies. The sensors can be embedded to form a variety of sensors, for instance, gas sensors, wearable sensors for ubiquitous monitoring of physiological parameters and other chronic diseases, a remote sensor, electrochemical sensor, real-time electronic monitoring sensor, piezoresistive pressure sensor, biosensor, strain sensor, human health medical monitoring sensor, optical sensor, and other published sensors. The development of sensors is highly vital to safeguard human health and environmental sustainability, upgrade recent technology, and in medical diagnosis, industrial manufacture, and national defence. The fabrication of graphene based polymeric nanocomposites enables that design of highly sensitive, reusable, low-cost, selective, and long-lasting gases sensing systems. A diverse range of topics have been discussed throughout this review to improve the quality of the sensor design, functionality and address the drawbacks of the previous sensors. The functionalised graphene–polymer sensors should be extensively studied and utilised to capture specific targeted molecules, especially NH_3_ molecules. As discussed in this paper, NH_3_ gas can harm the environment and human health, which necessitates the need for the development of a specific gas sensor.

There are many ways to improve the quality of sensor devices, such as using less complex and low input power devices, fabricating efficient sensors with consistent performance, and selecting appropriate materials and methods that consume less production cost with multiple distinctive parameters in a single sensing system. The addition of polymer into graphene not only enhances the sensor performance but the properties of the polymer itself, such as being lightweight, durable, flexible, and cheap, contribute to energy conservation and cost reduction. The challenges of graphene–polymer nanocomposites are associated with various parameters, such as the orientation of graphene layers, the type of graphene used, the preparation method, and the ratio of graphene to the polymer matrix. The use of graphene–polymer nanocomposites in improving gas sensors performance as well the methods used to fabricate graphene and graphene polymeric nanocomposites have been clearly explained in this review paper. The promotion of graphene–polymer nanocomposites as a NH_3_ gas sensor device has the biggest marketing industry with the prediction of all academician and corporates sectors to benefit the information of graphene–polymer nanocomposites-based ammonia gas sensor. The use of graphene–polymer nanocomposites is highly in demand and increasing with time, and it is expected to greatly impact the quality of life for the future generation. Based on the above discussions, more studies are still required to further study the interfacial interactions and synergetic effects that occur between graphene and polymer matrices which results in improving the sensing performance of gas sensors. In addition, ways achieve homogenous distribution of graphene nanofillers within the polymer matrices and the role of graphene fillers in improving the sensitivity of these sensors require further in-depth experimental studies.

## 10. Conclusions

The unique electrical, physical, and chemical properties of graphene and its hybrid nanomaterial-based sensors have driven the interest of researchers from all over the world in various fields. Graphene is easy to synthesise via notable methods, such as mechanical exfoliation, chemical or thermal reduction, CVD, epitaxial growth, and longitudinal “unzipping” of CNTs. GO can be produced from graphite as the precursor following Hummers’, Staudenmaeir’s, and Brodie’s methods. Likewise, polymer electrolyte has been introduced to a variety of applications, and the combination of graphene and polymers can enhance the ability of each material depending on the targeted application. A full explanation of the NH_3_ adsorbing mechanism has been reviewed and illustrated. Although various applications of graphene have been discovered and reported, there are still many aspects that need to be further explored, specifically the application and the potential of both graphene and its hybrid nanomaterials. In conclusion, the suitable selection of the methods and techniques for the synthesis of graphene and its hybrid nanomaterials affects the commercialisation of graphene as next-generation gas sensors.

## Figures and Tables

**Table 1:** Effects of ammonia exposure on human health [20].

Concentration of Exposure (ppm)	Health Hazard Conditions
35 (15 min of exposure)	Irritation to the respiratory tract, eyes, and skin; cell damage
53	Detectable odour
100	Tolerable exposure
450	Minor eyes irritation
2500–4500	Fatal within 30 min exposure
5000	Rapid respiratory arrest
10,000	Skin damage, conjunctivitis, and death

**Table 2:** Synthesis method of graphene and its derivatives (e.g., rGO).

Synthesis Method	Advantages	Limitations	Precursor	References
1) Mechanical exfoliation (Scotch tape)	High electronic quality of layers Low cost Forms single to multiple layers Size of layer: 10 µm	Low throughput Incompatible with the chip fabrication process Complicated Low probability of finding suitable individual graphene sheets Inapplicable at a large scale Not manageable	Graphite	[28,49,50]
2) Epitaxial growth by thermal desorption of silicon atoms	High electronic quality of layers Forms single to multiple layers High-quality graphene Size of layer: >50 pm Compatible with the chip fabrication process Good quality and more consistently graphene	High cost Low throughput Requires high vacuum conditions and specialised Expensive fabrication systems to generate only small-area films	SiC surface	[51,52,53]
3) Epitaxial growth by CVD on transition metals	High electronic quality of layers Low cost Forms single to multiple layers Size of layer: >100 µm	Low throughput Compatible with the chip fabrication process	Hydrocarbons	[54,55]
4) CVD	Large surface area Produce flat and smooth graphene High throughput Forms single to multiple layers Produce the best quality graphene Cost effective Manageable process	High resistivity Poor conductivity	GO	[56,57,58]
5) Longitudinal “unzipping” of CNTs	Affords large quantities of graphene nanoribbons, the width of which are dependent on the CNT diameter Scalable Abundant functional groups Facile Low cost	Incompetent as a stand-alone sensor device	CNTs	[58,59]
6) Reduction of graphene derivatives	Yields large amounts of graphene-like sheets Low cost	Not defect-free	GO	[60,61]
7) Liquid phase exfoliation	Size of layer: 100–1000 nm Forms single to multiple layers High-quality of graphene Very small fragment Low cost High throughput	Low electronic quality of layers	Graphite oxide	[62,63]

**Table 3:** The advantages and limitations of NH_3_ gas sensor fabrication methods.

Method	Advantages	Limitations	References
Sol-gel	Highly conductive High mobility Two-step reaction facilitates the incorporation of certain trace elements uniformly and quantitatively Uniform doping at the molecular level Easy chemical reaction Applicable under low temperatures Prepare various materials after varying the reaction parameter Affordable Short preparation Simple and economical Better controlled material structure Good sintering performance High powder purity Fine particles Simple equipment Convenience operation	High energy consumption Utilises a low-energy consumption method Density of the obtained gel greater than 0.1/cm^3^ Unsuitable for hydrophilic and brittle substrates Materials easy to agglomerate Large drying shrinkage Expensive cost of metal alkoxides Damage to health by organic matter Difficulty in industrial production	[101,102]
Hydrothermal/solvothermal	No aggregation occurrence High sensitivity even at RT Short response time Short saturation time More active sites provided	Unstable under certain humidity conditions High energy consumption	[103,104]
Layer-by-layer deposition	Open to a versatile assembly of polymers incorporating diverse building blocks Increase efficiency Enhance mechanical properties of capsules of the coated materials Extensive range of materials for sensors Many templates, such as planar and spherical Assembly is freely suspended in water (applicable for colloidal particles) Simple	Costly Time consuming Numerous biocompatibility issues	[105,106]
Template-assisted deposition	Low cost Easy synthesis without the removal of the template Applicable for large-scale production The materials can retain high crystallinity	Feeble quality distribution of the attained product Broad size distribution and varied size and shapes of the product Formation of internal stress structural defects and deterioration Require time and trials to determine the appropriate host materials	[107,108]
Physical vapour deposition	Pollution-free High purity substrate The source of vapourised material may be solid in any form High vaporisation rates Easy monitoring Affordable	Various alloy compositions and compounds can only be deposited with difficulty Line-of-sight and limited-area sources result in poor surface coverage on complex surfaces without proper fixturing and fixture movement Poor source material utilisation High radiant heat loads in the system	[109,110]

**Table 4:** Previous studies of graphene-based materials synthesised using the sol-gel method.

Composite	Analyte Gas	Operating Temperature (℃)	Concentration	Sensor Response (%)	T_res_/T_rec_ (s)	Reference
ZnO/CNT/SiO_2_ nanorods	H_2_	300	1000 ppm	66	-	[116]
Mg-doped ZnO thin films	Acetic acid	300	200 ppm	136	145/110	[111]
rGO/CoTiO_3_ nanosheets	Ethanol	195	50 ppm	9.03	2/5	[112]
Graphene/TiO_2_ nanoparticles	NO_2_	RT/UV	70–1750 ppb	1.17–3.14	35/90	[113]
rGO-Fe_3_O_4_ nanoparticles	NO_2_	200	2–5 ppm	4.68	-	[114]
rGO-Fe_3_O_4_ nanoparticles	Ethanol	RT	1 ppm	1.86	-	[114]

**Table 5:** Summary of previous studies of graphene-based materials synthesised using the hydrothermal/solvothermal method.

Composites	Analyte Gas	Operating Temperature (°C)	Concentration (ppm)	Sensor Response (%)	T_res_/T_rec_	Reference
ZnO nanoparticles decorated on 3D rGO	CO	200	1000	85.2	7 s/9 s	[117]
	CO	RT	1000	27.5	14 s/15 s	
Pd-doped SnO_2_/prGO nanocomposites	CH_4_	RT	14,000	0.5–10	5 min/7 min	[104]
SnO_2_ nanorods–nanoporous graphene hybrid	CH_4_	150	1000	24.9	369 s/-	[120]
3% rGO-In_2_O_3_ composites	NO_2_	74	1	1337	-	[121]
5% rGO-In_2_O_3_ composites	NO_2_	RT	1	1098	-	
rGO-TiO_2_ hybrid	NH_3_	RT	10	75	114 s/304 s	[122]
SnO_2_/rGO	Ethanol	250	100	77.1	9 s/457 s	[123]
2D nanostructured rGO/WS_2_ heterojunction	NH_3_	RT (20% RH)	10	121	60 s/300 s	[124]
rGO-CuO nanocomposites	NH_3_	150	600	<0.1	12 s/90 s	[125]
rGO/WO_3_	NH_3_	300	100	11	37 s/711 s	[126]
3D rGO/PANI hybrid	NH_3_	RT	50	10.8	370 s/675 s	[127]
Sn-TiO_2_@rGO/CNT	NH_3_	RT	250	85.9	99 s/66 s	[128]
Ppy-GO-WO_3_ hybrid nanocomposites	NH_3_	RT	10	58	50 s/120 s	[129]
1% GO.WO_3_ nanorods	NH_3_	200	100	17.6	10–15 s	[130]
Ppy/rGO	NH_3_	200	<1	6.1	1 min/5 min	[47]
rGO/WO_3_ nanocomposites	NH_3_	RT	10	4.35	13 s/20 s	[131]
	NH_3_	150	10	10.89	11 s/17 s	
	NH_3_	150	100	27.7	7 s/9 s	

**Table 6:** Previous studies of graphene-based materials synthesised using the layer-by-layer deposition method.

Composites	Analyte Gas	Operating Temperature (°C)	Concentration (ppm)	Sensor Response (%)	T_res_/T_rec_ (s)	Reference
rGO/PDDA	NH_3_	RT	50	5.7	24/805	[133]
Polyethene glycol/multiwalled CNTs	VOCs (acetone, ethanol, isopropanol, water)	RT	10–1000	0.0006	110/152	[134]
Poly(sodium-4-styrene sulfonate) (PSS)-graphene/polyallylamine hydrochloride (PAH) multilayer films	NO_2_	RT	5	66	-	[135]
In_2_O_3_ nanofibre/rGO	NH_3_	RT	15	95	17/214	[136]
GO/PAH	NH_3_	RT	5	68	68/274	[137]

**Table 7:** Summary of previous studies on graphene-based materials synthesised using the PVD method.

Composite Material	Analyte Gas	Operating Temperature (°C)	Concentration (ppm)	Sensor Response (%)	T_res_/T_rec_ (s)	Reference
Poly(3,4-ethylenedioxythiophene)-poly(styrenesulfonate) GO (PEDOT:PSS:GO)	NH_3_	RT	20	194	95/121	[151]
3D nitrogen-doped graphene-based framework/polyaniline (NiN_3_@3D-(N)GFs/PANI) hybrid	NH_3_	RT	1000	750.2	95/25	[152]
S and N co-doped graphene quantum dots/polyaniline (S,N GQDs)/PANI) hybrid	NH_3_	RT	100	42	115/44	[150]
Polyaniline/3D rGO hybrid films (PANI/3D rGO)	NH_3_	RT	5	111	35/187	[153]
Hollow carbon sphere polyvinylpyrrolidone (HCS/PVP)	NH_3_	20–40	74	46	>2000/<300	[154]
3D rGO/polyaniline (3D rGO/PANI) hybrid	NH_3_	RT	50	10.98	370/675	[155]

## Data Availability

Not applicable.
